# The phosphorylation of a WD40-repeat protein negatively regulates flavonoid biosynthesis in *Camellia sinensis* under drought stress

**DOI:** 10.1093/hr/uhae136

**Published:** 2024-05-05

**Authors:** Zhu Li, Yunyun Han, Xin Li, Jingjuan Zhao, Nana Wang, Yangyang Wen, Tongtong Li, Huangqiang Su, Liping Gao, Tao Xia, Yajun Liu

**Affiliations:** School of Life Science, Anhui Agricultural University, Hefei 230036, Anhui, China; School of Life Science, Anhui Agricultural University, Hefei 230036, Anhui, China; School of Life Science, Anhui Agricultural University, Hefei 230036, Anhui, China; Lu’an Institute of Product Quality Supervision and Inspection, Lu’an City, China; School of Life Science, Anhui Agricultural University, Hefei 230036, Anhui, China; Lu’an Institute of Product Quality Supervision and Inspection, Lu’an City, China; State Key Laboratory of Tea Plant Biology and Utilization / Key Laboratory of Tea Biology and Tea Processing of Ministry of Agriculture / Anhui Provincial Laboratory of Tea Plant Biology and Utilization, Anhui Agricultural University, Hefei 230036, Anhui, China; School of Life Science, Anhui Agricultural University, Hefei 230036, Anhui, China; School of Life Science, Anhui Agricultural University, Hefei 230036, Anhui, China; State Key Laboratory of Tea Plant Biology and Utilization / Key Laboratory of Tea Biology and Tea Processing of Ministry of Agriculture / Anhui Provincial Laboratory of Tea Plant Biology and Utilization, Anhui Agricultural University, Hefei 230036, Anhui, China; School of Life Science, Anhui Agricultural University, Hefei 230036, Anhui, China; State Key Laboratory of Tea Plant Biology and Utilization / Key Laboratory of Tea Biology and Tea Processing of Ministry of Agriculture / Anhui Provincial Laboratory of Tea Plant Biology and Utilization, Anhui Agricultural University, Hefei 230036, Anhui, China; School of Life Science, Anhui Agricultural University, Hefei 230036, Anhui, China; State Key Laboratory of Tea Plant Biology and Utilization / Key Laboratory of Tea Biology and Tea Processing of Ministry of Agriculture / Anhui Provincial Laboratory of Tea Plant Biology and Utilization, Anhui Agricultural University, Hefei 230036, Anhui, China

## Abstract

Flavonoids constitute the main nutraceuticals in the leaves of tea plants (*Camellia sinensis*). To date, although it is known that drought stress can negatively impact the biosynthesis of flavonoids in tea leaves, the mechanism behind this phenomenon is unclear. Herein, we report a protein phosphorylation mechanism that negatively regulates the biosynthesis of flavonoids in tea leaves in drought conditions. Transcriptional analysis revealed the downregulation of gene expression of flavonoid biosynthesis and the upregulation of *CsMPK4a* encoding a mitogen-activated protein kinase in leaves. Luciferase complementation and yeast two-hybrid assays disclosed that CsMPK4a interacted with CsWD40. Phosphorylation assay *in vitro,* specific protein immunity, and analysis of protein mass spectrometry indicated that Ser-216, Thr-221, and Ser-253 of CsWD40 were potential phosphorylation sites of CsMPK4a. Besides, the protein immunity analysis uncovered an increased phosphorylation level of CsWD40 in tea leaves under drought conditions. Mutation of the three phosphorylation sites generated dephosphorylated CsWD40^3A^ and phosphorylated CsWD40^3D^ variants, which were introduced into the Arabidopsis *ttg1* mutant. Metabolic analysis showed that the anthocyanin and proanthocyanidin content was lower in *ttg1:CsWD40^3D^* transgenic plants than *ttg1::CsWD40^3A^* transgenic and wild type plants. The transient overexpression of *CsWD40^3D^* downregulated the anthocyanidin biosynthesis in tea leaves. The dual-fluorescein protein complementation experiment showed that CsWD40^3D^ did not interact with CsMYB5a and CsAN2, two key transcription factors of procyanidins and anthocyanidins biosynthesis in tea plant. These findings indicate that the phosphorylation of CsWD40 by CsMPK4a downregulates the flavonoid biosynthesis in tea plants in drought stresses.

## Introduction

Drought is one of the most severe natural hazards and has been reported to lead to more than 50% yield loss of food and economic crops [1, 2]. Tea plant (*Camellia sinensis*) is a global beverage woody crop. It is rich in flavonoids, which are associated with the quality of health benefits and flavor of tea products. The main tea flavonoids include catechins, flavonols, proanthocyanidins, and anthocyanins, accounting for 12–24% of the dry weight of tea leaves [3]. To date, the biosynthesis of these compounds has gained intensive studies in different plants. A regulatory complex has been elucidated to consist of myeloblastosis (MYB) and helix–loop–helix (bHLH) and a WD-repeat protein (WD40), which regulates the biosynthesis of anthocyanins and proanthocyanidins [4]. In tea plants, a MBW complex has been characterized to include CsMYB5a, CsTT8 (TRANSPARENT TESTA 8), and CsWD40, which primarily regulates the biosynthesis of flavanols. Another MYB complex consisting of CsMYB6a, CsTT8, and CsWD40 has been characterized to regulate the biosynthesis of anthocyanins [5]. In these complexes, the WD-repeat proteins are characterized with the presence of 5~8 WD motifs. The WD-repeat proteins are a diverse family that acts as platforms for the assembly of protein complexes or mediators of transient interactions among other proteins. Previous reports have shown that the WD family plays essential roles in various aspects of plant cell physiology, development, as well as in response to and defense against environmental stresses [6].

WD40 encoded by *TTG1 *in *Arabidopsis* and its homologs in other plants has been shown to be involved in seed development, post-embryonic development, trichome formation on vegetative organs, root hair development, and phenolic metabolism regulation [7–9]. In addition, experimental evidence suggests that WD40 is involved in the regulation of plant response to abiotic stresses [10], such as drought, salt, and high sugar treatment. The complementary expression of *SiTTG1* from *Setaria italica* in the Arabidopsis *ttg1–13* mutant was reported to increase the expression levels of stress-responsive genes in the transgenic seedlings and the resistance of plants to salinity and high glucose stresses during germination and seedling establishment [11]. Another study has reported that the complementary expression of *LbTTG1* from *Limonium bicolor* in the Arabidopsis *ttg1–13* mutant increased salt tolerance by reducing ion accumulation and enhancing osmolyte levels [12].

**Figure 1:**
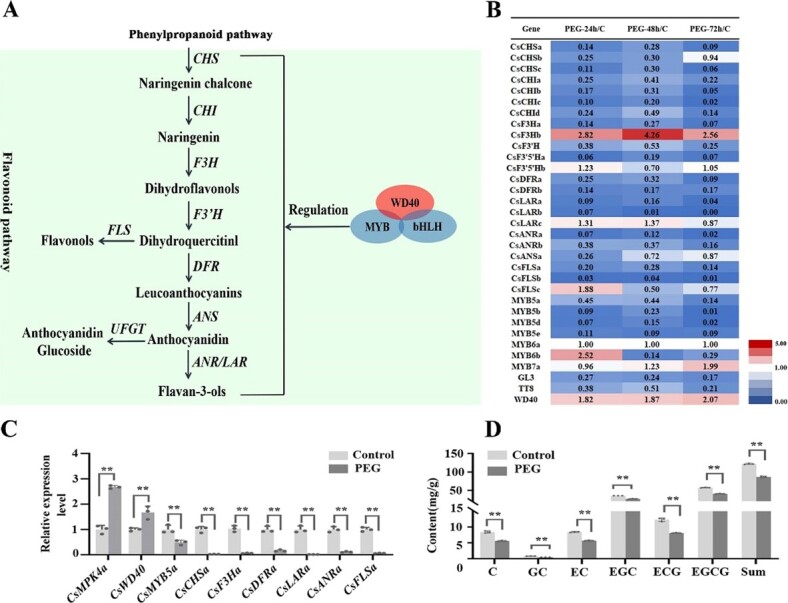
Effects of drought stress on the biosynthesis of flavonoid in tea leaves. **A** Diagram of flavonoid biosynthetic pathway. **B** Expression level of structural and key transcription regulatory genes in flavonoid pathway of tea plant under drought stress by 20% PEG4000 for 24 h, 48 h, 72 h. The FPKM values of gene expression at different time points are listed in Data S3 (see online supplementary material). **C** The expression levels of key genes in the flavonol and catechin biosynthesis pathways in tea leaves were verified by qRT-PCR under drought stress at 48 h. The gene expression level data from 0–48 hours are listed in Fig. S2 (see online supplementary material). **D** The catechin content in tea leaves under drought stress at 48 h. The relative variation in phenolic compound content during the 0–48 h drought treatment is shown in Data SS1.

The mitogen-activated protein kinases (MAPKs) are a protein kinase family that is specific to the phosphorylation of serine and threonine of proteins. The MAPKs cascade is involved in transducing extracellular stimuli into intracellular responses in eukaryotic organisms. A typical MAPK cascade involves three different types of kinases: MAPKK kinases (MAPKKKs; also known as MAP3Ks or MEKKs), MAPK kinases (MKKs; also known as MAP2Ks or MEKs), and MAP kinases (MAPKs; also known as MPK) [13]. MAPKs can be divided into A, B, C, and D groups. Groups A, B, and C contain a TEY motif, while group D contains a TDY motif. Arabidopsis MPK3 and MPK6, two members of group A have been reported to mainly participate in plant defense responses to pathogens, and are also involved in response to abiotic stress, plant growth, and development [14]. Arabidopsis MPK4, MPK5, MPK11, MPK12, and MPK13 in group B have been shown to play important roles in plant immunity, response to environmental changes, growth, and development [13]. In addition, Arabidopsis MPK1, MPK2, MPK7, and MPK14 classified in group C remains for functional analysis. In Arabidopsis, different MAPK pathways have been disclosed to participate in various signaling transductions. The MAPK signaling pathway MEKK1-MKK1-MPK4 in Arabidopsis is characterized to involve in transmitting signals related to wounding, cold, drought, and high salt stress [15]. The GhMPK31 (orthologous to MPK4 in Arabidopsis) signaling pathway has been shown to regulate drought stress tolerance in cotton [16]. According to the publicly available TPIA database (http://tpia.teaplant.org/), we identified 16 MAPK members in tea plants. Although the function of these members remains unknown, a transcriptomic report suggested that these MAPKs might be involved in the response of tea plants to cold, drought, and salt stress [17].

*C. sinensis* is native to Southwest China and widely cultivated in tropical and subtropical areas. Because of its native habitat, drought is a common natural disaster in tea plant growth and changes metabolism in tea leaves [18]. A previous study has reported a significant reduction in the accumulation of flavonoid compounds and the expression of their biosynthetic genes in *C. sinensis* leaves under a long-term drought stress [19]. To understand mechanisms associated with this reduction of flavonoid metabolism under drought conditions, we used an integrative approach to identify genes and characterize their functions in tea leaves.

## Results

### Flavonoid biosynthesis reduced in *C. sinensis* leaves under drought stress

Flavonoids are the predominant secondary metabolites in leaves of *C. sinensis*. The flavonoid and lignin pathways are two downstream branches of the phenylpropanoid pathway (Fig. 1A; Fig. S1A, see online supplementary material). Data mining was performed with transcriptomes obtained from drought-treated tea plants grown in polyethylene glycol (PEG) conditions. The results indicated that the expression of genes involved in phenylpropanoid, flavonoid, and lignin biosynthesis was altered in a prolonged drought treatment [20, 21]. After 72 hours of drought treatment, the gene expression in the phenylpropanoid and lignin pathways was upregulated, while the expression of genes associated with flavonoid biosynthesis was downregulated in leaves (Fig. 1B; Fig. S1B, see online supplementary material). *CsDFRa* (DFR, dihydroflavonol reductase), *CsLARa* (LAR, leucoanthocyanidin reductase), and *CsANRa* (ANR, anthocyanidin reductase) are three pathway genes of flavan-3-ols. The expression levels of *CsDFR*, *CsLARa*, and *CsANRa* were reduced by 10%, 96%, and 98% compared with controls (Fig. 1B). R2R3-MYB, bHLH, and WD40 proteins form MBW complexes, which regulate multiple pathway genes in the flavonoid biosynthesis [4]. In tea plants, *CsMYB5a*, *CsMYB5b*, *CsMYB5d*, and *CsMYB5e* are four R2R3-MYB members, *CsGL3* (GLABROUS3) and *CsTT8* are two bHLH members. Their expression levels were downregulated in leaves of PEG-treated plants (Fig. 1B). By contrast, the expression level of *CsWD40* was upregulated in leaves by the PEG treatment.

Based on the above-mentioned transcriptomic data, tea shoots were inoculated on ½ MS medium supplemented with 20% PEG. The RT-qPCR results showed that the expression levels of the flavonoid pathway genes exhibited a reduction trend in the treatment group compared to the control group (Fig. S2, see online supplementary material). Especially, the expression levels of *CsFLS*, *CsLAR*, and *CsANR* were decreased by 90% compared to the control after 48 hours of PEG treatment (Fig. 1C). These results supported the gene expression profiles (Fig. 1A) observed in transcriptomes described above. LC–MS analysis was carried out to measure flavonoids. The results revealed a decreasing trend in the content of flavonoids in the 20% PEG-treated samples compared with the controls (Data SS1, see online supplementary material). Quantification showed that after 48 hours’ treatment, the content of total catechins was 86.01 mg/g in the treated leaves and 122.12 mg/g in leaves of the control group (Fig. 1C; Data S2, see online supplementary material), indicating that the treatment led to approximately 30% reduction of total catechins. In further experiments, phenolic compound variation in plants under non-irrigation treatment was consistent with the results of PEG treatment (Data S3, see online supplementary material).

**Figure 2:**
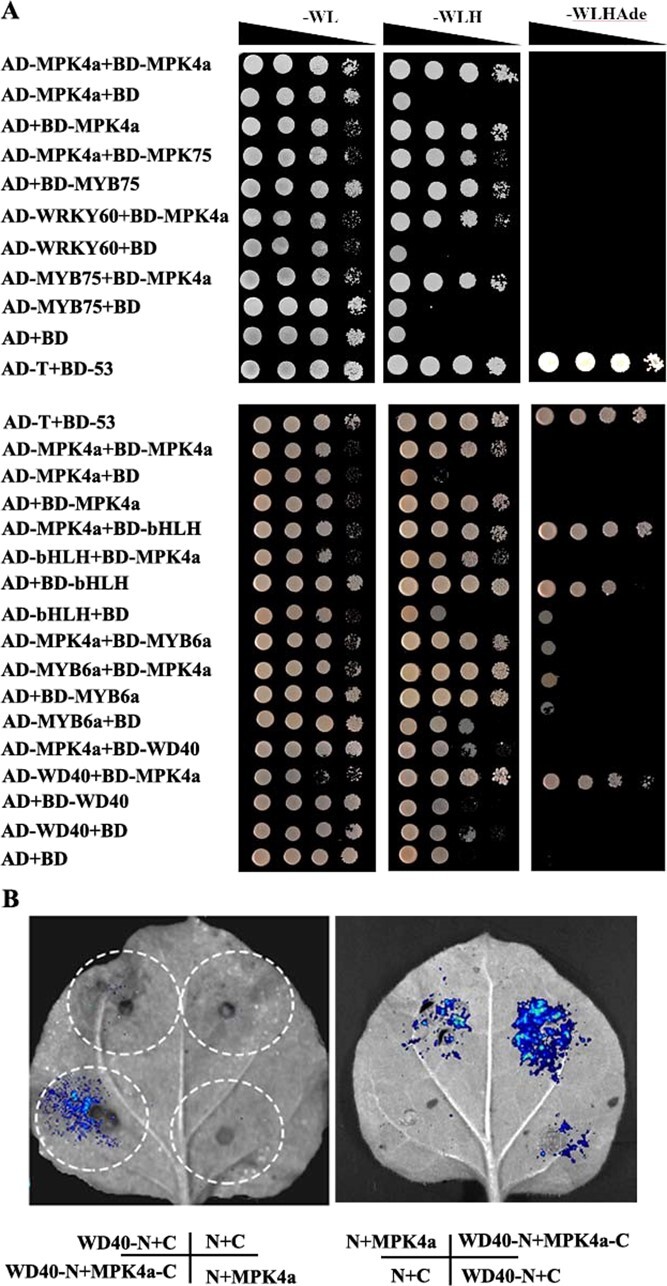
Interaction between CsMPK4a and proteins regulating the flavonoid biosynthesis by Yeast two-hybrid and dual luciferase assay. **A** Screening of CsMPK4a interacting proteins involved in regulation of flavonoid biosynthesis in tea plants. **B** A dual luciferase assay in *N. benthamiana* leaves shows that CsWD40 interacts with CsMPK4a. MPK4a-C represents CsMPK4a-cLUC; WD40-N, CsWD40-nLUC; C, cLUC empty; N, nLUC empty. –WL, yeast growth on medium without Trp and Leu. –WLHAde, yeast growth on medium lacking Trp, Leu, His, and Adenine.

### Screening of kinase involved in the regulation of tea flavonoid biosynthesis

MAPK cascades act as a molecular switch in sensing upstream signaling and responding to environmental stresses [22]. Sixteen MPK kinases were identified from the genome and transcriptome of tea plants (Fig. S3, see online supplementary material). Based on the conserved motifs of ‘TEY’, ‘TDY’, they were classified into Group A–D (Fig. S3A, see online supplementary material). Most of the drought-induced *MPK* genes were classified into groups D and B, such as *CsMPK4a-b*, *CsMPK9a-c*; while *MPK* gene in group C exhibited an overall downregulation trend (Fig. S3B, see online supplementary material). Among them, *CsMPK4a* exhibited the most significant drought-induced expression, with its expression level being above five times higher than that of the control at 48 h. The sequence identity between CsMYB4a and CsMYB4b is high. However, MYB4b lacks nine amino acids at the N-terminus, and its functional regulation is weaker than MYB4a (data not shown). As a result, CsMYB4a was chosen for subsequent in-depth research in this work.

**Figure 3:**
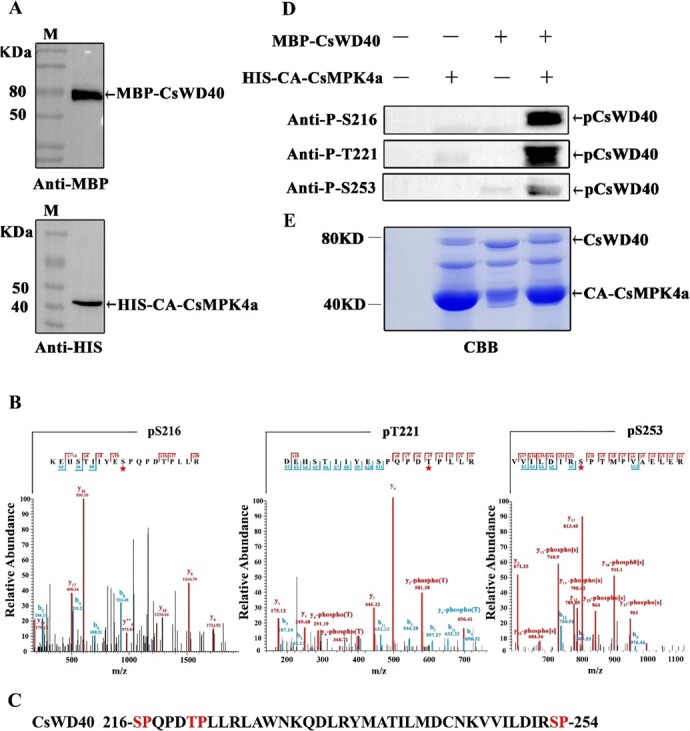
Identification of CsMPK4a-mediated phosphorylation sites on CsWD40 *in vitro.*  **A** Prokaryotic expression proteins of CA-CsMPK4a and verification by immune blot with His and MBP antibodies. **B** LC–MS/MS spectrum of the CsWD40 peptide containing serine 216, threonine 221, serine 253 phosphorylated by CsMPK4a. **C** Schematic diagram of the sites on CsWD40 protein potentially phosphorylated by CsMPK4a. **D** Phosphorylation reaction between CA-CsMPK4a and CsWD40 *in vitro*, and immune blot with anti-P-S216, anti-P-T221 and anti-P-S253 bodies. **E** CsWD40 and CsMPK4a in the enzyme reaction solution were identified by Coomassie blue staining.

Yeast two-hybrid (Y2H) system was used to screen the potential target protein of CsMPK4a involved in flavonoid biosynthesis. The proteins CsMYB75 (CsMYB6a), CsWRKY60, CsbHLH, CsWD40 were selected to perform one-to-one interaction with CsMPK4a. The results indicated CsMPK4a interacted with CsWD40 (Fig. 2A). Because the control yeast also grew on the defective medium, it cannot be determined whether CsbHLH (CsTT8) interacts with CsMPK4a. It is reported that AtMPK4 interacted with AtMYB75 and phosphorylated it [23]. The interaction between CsMPK4a and CsMYB75 was performed, but the results were negative. The protein sequence alignment showed CsMYB75 lacked the phosphorylation site of MPK kinase (Fig. S4, see online supplementary material).

The kinase activity of MPK depends on its own phosphorylation, and its interaction with downstream substrate proteins is probably generally transient [24]. To mitigate the impact of this deficiency on Y2H interaction, we constructed a constitutively active form of CsMPK4a (CA-CsMPK4a), and performed the above protein interaction; the result was the same (Fig. S5, see online supplementary material). That indicated phosphorylation of CsMPK4a itself does not affect its interaction with target proteins. The interaction between CsMPK4a and CsWD40 in plant was further validated by dual luciferase assay in *Nicotiana benthamiana*. The experimental results showed that fluorescence was observed in the experimental group co-expressing CsMPK4a-cLUC and CsWD40-nLUC in the same leaf, while no obvious fluorescence was observed in the control group co-expressing CsMPK4a-cLUC and nLUC empty vector, cLUC empty vector, and CsWD40-nLUC. These results indicated that CsMPK4a- cLUC and CsWD40-nLUC can interact with each other in planta, which further confirmed the results of the yeast two-hybrid experiment mentioned above.

### Identification of CsMPK4a-mediated phosphorylation sites in CsWD40

Based on the structural characteristic, the CsWD40 sequence is predicted to contain five phosphorylation sites of CsMPK4a (Fig. S6, see online supplementary material). To identify the main phosphorylation sites mediated by CsMPK4a, the phosphorylation assay *in vitro* was performed. CsWD40 and CsMPK4a were fused with the protein tag His and MBP, respectively, and expressed in *Escherichia coli*. The recombinant proteins of CsWD40 and CsMPK4a were purified and verified by immune blot with His and MBP antibodies (Fig. 3A). The enzymatic products were identified by LC–MS/MS. The results showed that three amino sites of CsWD40, serine 216, threonine 221, serine 253, were phosphorylated (Fig. 3B). In order to further confirm this result, we prepared phospho-specific antibody against these three sites on CsWD40. The results showed each phospho-specific antibody recognized their cognate residues, demonstrating that serine 216, threonine221, serine 253 in CsWD40 were phosphorylated

**Figure 4:**
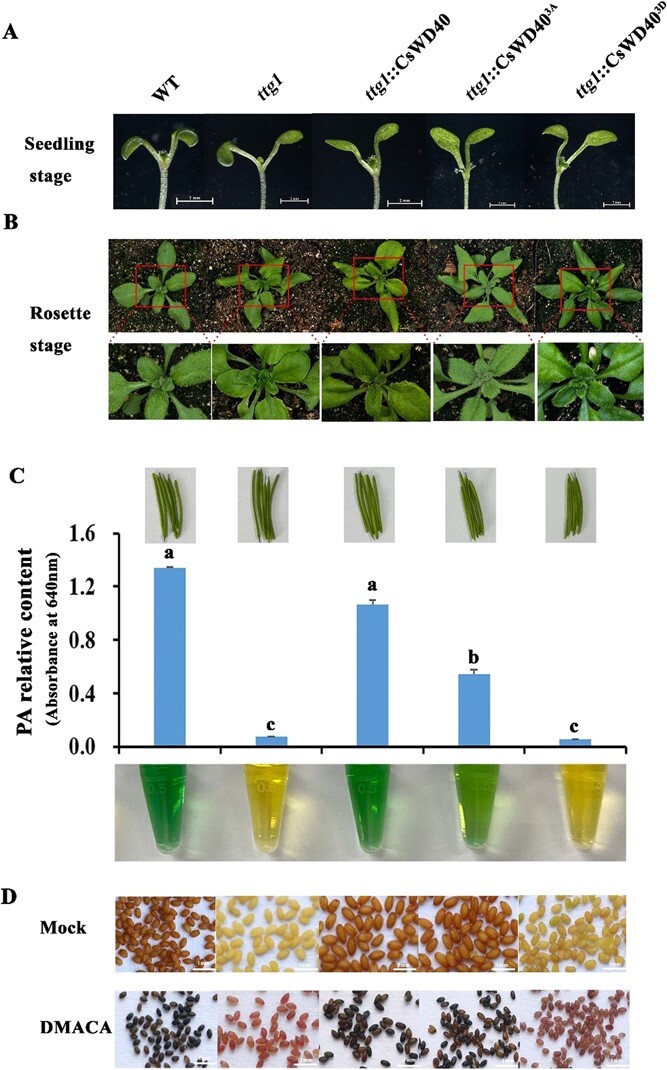
Phenotype and proanthocyanidin content analysis of tri-site phosphorylated and dephosphorylated CsWD40 transgenic *Arabidopsis thaliana.*  **A** Phenotype of *ttg1*::*CsWD40*^3A^ and *ttg1*::*CsWD40*^3D^ transgenic *A. thaliana* plants at seedling stage. **B** Phenotype of the transgenic *A. thaliana* at the rosette stage. **C** Proanthocyanidin content in green fruit pod of the transgenic plants. **D** The color of seed coat and proanthocyanidin accumulation of transgenic mature seeds. The accumulation of proanthocyanidins in the seeds can be visualized by staining with DMACA.

 by CsMPK4a (Fig. 3D).

### Functional analysis of the dephosphorylated and phosphorylated CsWD40 variants in Arabidopsis *ttg1* mutant

In order to investigate the functional role of CsWD40 phosphorylation, we generated mutants *ttg1::CsWD40^3A^* through dephosphorylation at three specific sites (serine 216, threonine 221, and serine 253), and corresponding *ttg1::CsWD40^3D^* mutants through phosphorylation at the same sites (Fig. S7, see online supplementary material).

The mutated genes were constructed into overexpression vectors driven by the 35S promoter and genetically transformed into Arabidopsis *ttg1* mutants, which have a null function of WD40. Compared with wild-type plants, *ttg1* mutants grow slower and lack trichomes on leaves and stems, and show weaker capacity of biosynthesizing anthocyanins and proanthocyanidins. Transgenic plants expressing *ttg1::CsWD40^3A^* and *ttg1::CsWD40^3D^* showed growth and developmental patterns similar to those of wild-type plants during the seedling and rosette stages. Additionally, they successfully reinstated trichome development on both leaves and stems (Fig. 4A and B). This suggests that dephosphorylation and phosphorylation of CsWD40 at the three amino acid residues do not affect the growth and trichome formation of transgenic Arabidopsis.

At the green pod stage of plants, the proanthocyanidins in pods were measured. The proanthocyanidin content in *ttg1::CsWD40^3D^* was low and close to that in the mutant, whereas the proanthocyanidin content in *ttg1::CsWD40^3A^* mutants was higher compared to that in *ttg1::CsWD40^3D^* mutants and the mutant, but lower than that in the wild type and the *ttg1::CsWD40* (Fig. 4C).

At maturity, the seed coat color of transgenic Arabidopsis *ttg1::CsWD40* and *ttg1::CsWD40^3A^* was deeper than that of the mutant *ttg1* and similar to that of the wild type, while the seed coat color of *ttg1::CsWD40^3D^* was similar to that of the mutant (Fig. 4D). Staining mature seeds with DMACA confirmed further that the proanthocyanidin content in the seed coat of *ttg1::CsWD40^3D^* mutant was similar to that of the *ttg1* mutant (Fig. 4D). Taken together, sustained phosphorylation of the three amino acid residues affected the accumulation of proanthocyanidins in the seed coat of transgenic Arabidopsis *ttg1::CsWD40^3D^*.

To identify which site plays a dominant role, we constructed single-site sustained phosphorylation mutants *ttg1::CsWD40^216D^*, *ttg1::CsWD40^221D^*, and *ttg1::CsWD40^253D^* (Fig. S7, see online supplementary material). During the seedling stage, no significant differences in phenotypes were observed among single-site phosphorylation mutation transgenic Arabidopsis lines and the wild type (Fig. S8A, see online supplementary material). At the rosette stage, obvious trichome formation was found in all three transgenic Arabidopsis plants (Fig. S8B, see online supplementary material), indicating that single-site sustained phosphorylation mutations in CsWD40 did not show an obvious influence on the trichome formation in transgenic Arabidopsis.

During the green pod stage, the proanthocyanidin content in *ttg1::CsWD40^216D^*, *ttg1::CsWD40^221D^*, and *ttg1::CsWD40^253D^* transgenic Arabidopsis plants was significantly higher compared to that in the ttg1 mutant, but lower than that in the wild type and the *ttg1::CsWD40* (Fig. S8C, see online supplementary material). At the mature seed stage, the seed coat color of *ttg1::CsWD40^216D^*, *ttg1::CsWD40^221D^*, and *ttg1::CsWD40^253D^* transgenic Arabidopsis plants appeared gray-brown and the color was similar to that of the *ttg1::CsWD40* and the wild type. DMACA staining revealed a high accumulation of proanthocyanidin in the seed coat of all three transgenic Arabidopsis plants (Fig. S8C, see online supplementary material). In summary, single-site phosphorylation mutations in CsWD40 had a limited impact on proanthocyanidin accumulation in Arabidopsis.

Methyl jasmonate or jasmonic acid induces anthocyanin accumulation significantly in Arabidopsis [25]. To further understand the impact of WD40 phosphorylation on anthocyanin biosynthesis in transgenic Arabidopsis, transgenic seedlings were treated with methyl jasmonate (MeJA). Wild-type, *ttg1::CsWD40^3A^* and *ttg1::CsWD40* transgenic Arabidopsis seedlings had high anthocyanidin accumulation in the petiole and stem under MeJA treatment, while *ttg1::CsWD40^3D^* transgenic Arabidopsis had a small amount of anthocyanidin accumulation (Fig. S9, see online supplementary material). These results suggested that phosphorylation of the three sites in CsWD40 impacted anthocyanidin accumulation in transgenic Arabidopsis under stress. Furthermore, when treated with MeJA, the primary roots of *ttg1::CsWD40^3D^* transgenic Arabidopsis were significantly longer than those of the wild type, *ttg1::CsWD40^3A^*, and *ttg1::CsWD40* transgenic plants (Fig. S10, see online supplementary material). This indicates that phosphorylation of the three sites on CsWD40 might mitigate the sensitivity to MeJA stress.

To elucidate the primary role of these phosphorylated sites, *ttg1::CsWD40^216D^*, *ttg1::CsWD40^221D^*, and *ttg1::CsWD40^253D^* transgenic seedlings were treated with MeJA. After six days’ treatment, all these three transgenic plants exhibited noticeable red color in the petiole and stem (Fig. S11, see online supplementary material). Analysis of the anthocyanin content revealed that the single-site-phosphorylated transgenic Arabidopsis plants had lower levels of anthocyanin accumulation compared to the *CsWD40* transgenic plants.

In combination, single mutations at positions 216, 221, and 253 had a limited impact on the accumulation of both anthocyanin and proanthocyanidin, while simultaneous phosphorylation of all these three sites decreased the accumulation of both anthocyanin and proanthocyanidin significantly in transgenic Arabidopsis.

### Mechanism of the CsWD40 phosphorylation in regulating anthocyanin and proanthocyanidin biosynthesis

WD40 proteins exert transcriptional activity in the nucleus of plants by forming trimeric complexes with bHLH and MYB transcription factors [26]. Does phosphorylation change the subcellular localization of CsWD40? The subcellular localization of CsWD40 protein after phosphorylation and dephosphorylation was verified using tea and Arabidopsis protoplast transient expression system. The results showed that CsWD40, CsWD40^3A^, and CsWD40^3D^ were all located in both the nucleus and cytoplasm (Fig. S12, see online supplementary material). This indicates that dephosphorylation or phosphorylation of CsWD40 does not affect its subcellular localization.

CsMYB5a and CsAN2 play a dominant role in the regulation of flavan-3-ol (catechin and proanthocyanidin) and anthocyanin biosynthesis in tea plants by forming trimeric complexes with CsTT8 and WD40, respectively [5, 27]. The interaction between CsWD40, CsWD40^3A^, and CsWD40^3D^ with CsMYB5a and CsAN2 was investigated using bimolecular fluorescence complementation (BiFC) in Arabidopsis protoplasts. Fluorescence signals localized in the nucleus were observed through co-expression of nYFP-CsWD40 and CsMYB5a-cYFP, CsMYB5a-cYFP and nYFP-CsWD40^3A^, and CsAN2-cYFP and nYFP-CsWD40^3A^. No YFP fluorescence signal was detected in protoplasts co-expressing CsMYB5a-cYFP and nYFP-CsWD40^3D^, or CsMYB5a-cYFP and nYFP-CsWD40^3D^. These findings suggest that phosphorylation of residues at the three sites of CsWD40 hinders its interaction with CsMYB5a and CsAN2 in plants.

**Figure 5:**
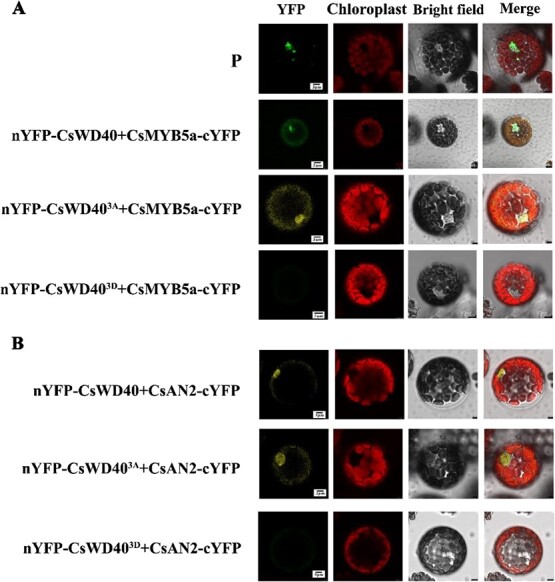
The effect of CsWD40 phosphorylation on the interaction between CsWD40 and CsMYB5a, CsAN2 was assessed through BiFC assay. **A** and **B** The interaction between CsWD40, CsWD40^3A^, and CsWD40^3D^ with CsMYB5a (**A**) and CsAN2 (**B**) was examined by BiFC assay in *Arabidopsis thaliana* protoplasts. P in the figure is the positive control group.

### The role of WD40 phosphorylation in the regulation of flavonoid biosynthesis in *C. sinensis*

The phosphorylation levels of CsWD40 at three sites were evaluated in tea shoot leaves exposed to drought treatment. Western blot analysis revealed an increase in the intensity of the phosphorylated CsWD40 band in tea samples treated with PEG for 48 hours compared to the control (Fig. S13, see online supplementary material). Conversely, the intensity of the non-phosphorylated CsWD40 band decreased, suggesting an elevated level of CsWD40 phosphorylation during PEG treatment.

**Figure 6:**
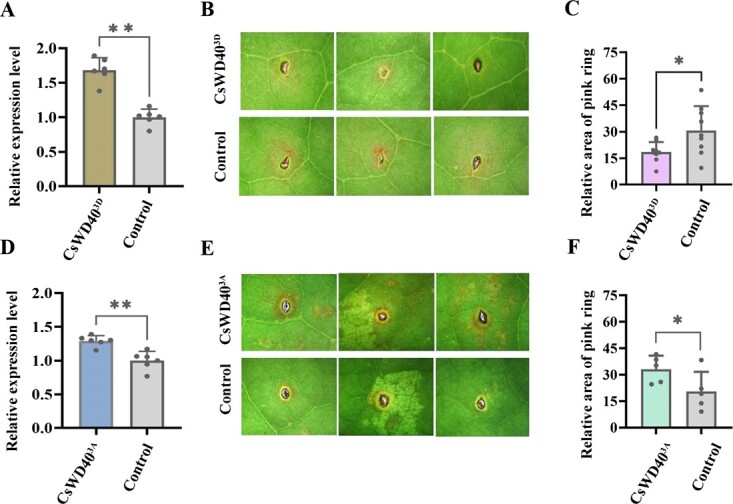
The dephosphorylation of CsWD40 negatively regulates anthocyanin biosynthesis in tea plants under stress. **A** and **D** Transgene verification detected by qRT-PCR. **B** Effect of transient overexpression of *CsWD40^3D^* on anthocyanin biosynthesis in tea leaves. **C** The relative area of pink ring in control group and treatment group of transient overexpression of *CsWD40^3D^*. **E** Effect of transient overexpression of *CsWD40^3A^* on anthocyanin biosynthesis in tea leaves. **F** The relative area of pink ring in control group and treatment group of transient overexpression of *CsWD40^3A^*. The anthracnose infection-induced anthocyanin biosynthesis system was employed to examine the effect of overexpressing the *CsWD40^3D^* and *CsWD40^3A^* gene on anthocyanin biosynthesis in tea leaves.

Our previous research has shown that the infection of Colletotrichum can induce the accumulation of anthocyanins in tea leaves [28]. In this study, we utilized this experimental system to investigate the impact of overexpressing the *CsWD40^3D^*, *CsWD40^3A^*, and *CsMPK4a* gene on anthocyanin biosynthesis in tea leaves. The transient overexpression of the *CsWD40^3D^*, *CsWD40^3A^*, and *CsMPK4a* genes in fresh leaves was confirmed through qRT-PCR analysis (Fig. 6A and D; Fig. S14A, see online supplementary material). Leaves with transient overexpression of the *CsWD40^3D^* gene exhibited a significant reduction in anthocyanin area (pink area) and intensity after Colletotrichum inoculation, in contrast to the control group (Fig. 6B and C). The transient overexpression of *CsMPK4a*, exhibits similar results to that of *CsWD40^3D^*, negatively regulating the biosynthesis of anthocyanins (Fig. S14, see online supplementary material). Conversely, leaves with transient overexpression of the *CsWD40^3A^* gene showed a more prominent increase in pink area compared to the control group (Fig. 6D and E). These results indicate that the expression of the *CsWD40^3D^* gene interferes with anthocyanin biosynthesis. In summary, under drought conditions, the expression levels of the *CsWD40* gene and protein increase, accompanied by an elevation in CsWD40 phosphorylation. Phosphorylation at three specific sites of CsWD40 inhibits anthocyanin biosynthesis in tea plants.

## Discussion

Under drought stress, plants enhance their drought signaling, synthesize and accumulate osmoprotectants, as common physiological responses [29]. Phenolics, which are major secondary metabolites synthesized by plants, are widely distributed throughout the plant kingdom [30]. Plants exhibit remarkable variations in the synthesis and accumulation of phenolic compounds in response to drought stress. Some plants, such as leaves and stems of *Arabidopsis thaliana* and maize, produce flavonoid compounds to improve drought tolerance [29, 31]. Flavonoids enhance drought tolerance by scavenging oxygen free radicals and mitigating stress-induced oxidative damage [32]. In plants with a high accumulation of organ-specific polyphenols, the biosynthesis of phenolic compounds may not be induced by drought. In soybean seeds, prolonged drought stress results in a significant reduction in total isoflavones [33]. In *Vitis vinifera*, both leaves and roots showed a significant reduction in total phenolic compounds and phenolic acid content under long-term drought stress [34]. Additionally, rutin concentrations in the leaves of *Bupleurum chinense DC* decreased by 38.79% and 30.11% during vegetative and reproductive growth stages, respectively, under drought treatment [35]. Excessive accumulation of phenolic substances in plants probably impacts their resistance to long-term drought pressure. Plants may prioritize biosynthesis by preferentially synthesizing more effective osmoprotectants to counteract drought stress through readjusting carbon allocation.

Tea plants are known for their high accumulation of polyphenols in leaves. It is reported that the onset of drought or mild drought within a certain range induces the synthesis of total polyphenols, possibly related to the accumulation of reactive oxygen species (ROS). However, as the drought period extends or intensifies, it leads to a decrease in the accumulation of total polyphenols or total flavonoids [36]. Jeyaramraja *et al.* investigated the impact of soil moisture variation on the content of catechins in pot-grown tea plants [37]. Both drought-tolerant and drought-sensitive cultivars exhibited a significant decrease in the major catechin components in fresh leaves under drought conditions. After 40 days of drought stress on nine adult tea plants grown on the field, the levels of tea polyphenols decreased [38]. Wang *et al.* subjected pot-grown tea plants to PEG treatment and conducted gene expression analysis at the early stage of drought, along with measuring the catechin content at 0, 2, and 5 days [19]. Genes related to flavonoid synthesis exhibit an initial increase followed by a decrease trend. The analysis of catechin content revealed a significant decrease in EC, EGC, EGCG, and ECG after 2 and 5 days’ drought treatment. The content detection results are consistent with ours obtained from PEG treatment on tea plant shoots in this work (Fig. 1).

Obviously, when tea plants are subjected to continuous drought stress, the biosynthesis of flavonoids significantly decreases. The lignin pathway and flavonoid pathway are two branches of the phenylpropanoid pathway. Analysis of gene expression patterns suggests that during drought stress in tea plants, lignin synthesis is significantly enhanced while flavonoid synthesis is inhibited (Fig. 1; Fig. S1, see online supplementary material). This observation indicates that tea plants, when faced with limited carbon supply during drought stress, prioritize the synthesis of lignin over flavonoids.

Kinase-mediated protein phosphorylation represents highly conserved signaling transduction modules and plays a central role in the transduction of various extra- and intracellular signals throughout plants. Two SHAGGY-like kinases 11/12 (SK11/SK12) phosphorylate TTG1 at a specific site, Ser215, in Arabidopsis. This phosphorylation enhances fatty acid biosynthesis in the embryo while inhibiting the production of mucilage and flavonoid pigments in the seed coat [39]. Phosphorylation of TTG1 at Ser215 abolishes its interaction with TT2, which encodes a R2R3-MYB transcription factor that acts as a key determinant in the proanthocyanidin accumulation of Arabidopsis seed. Amino acid sequence alignment revealed that the Ser215 residue in the AtWD40 protein corresponded to the Ser216 residue in the CsWD40 protein, which was one of the sites of interest in this study (data not shown). A MAPK kinase, AtMPK4 regulates UV-induced anthocyanin synthesis by phosphorylating MYB75 in Arabidopsis. However, CsMPK4a, the ortholog of AtMPK4 in tea plants, showed no significant induction under UV treatment. Additionally, CsMYB75, did not have any phosphorylatable TP or SP sites for CsMPK4a phosphorylation (Fig. S4, see online supplementary material). Yeast two-hybrid experiments confirmed that they did not interact with each other (Fig. 2). Interestingly, CsMPK4a in tea plants was induced by drought stress. Luciferase complementation imaging and yeast two-hybrid analyses indicated the interaction between CsWD40 and MPK4a. CsMPK4a can phosphorylate CsWD40 at residues 216, 221, and 253 in phosphorylation assays *in vitro*. Transgenic Arabidopsis experiments demonstrated that phosphorylation of these three residues in CsWD40 affected the accumulation of anthocyanins and proanthocyanidin in plants and seeds, respectively.

The dephosphorylation mutations at these three sites had a certain level of effects on the accumulation of proanthocyanidins in transgenic Arabidopsis at the green pod stage. In the *ttg1::CsWD40^3A^*, *ttg1::CsWD40^216A^*, and *ttg1::CsWD40^221A^* transgenic Arabidopsis lines, the proanthocyanidin content was significantly lower than that of the control groups of the wild-type and *ttg1::CsWD40* during pod development. However, at the mature stage, DMACA staining results indicated no significant difference in seed proanthocyanidin content compared to the wild-type and *ttg1::CsWD40^3D^* control groups. The WD40 protein (TTG1) in Arabidopsis regulates the accumulation of seed storage proteins and fatty acids during seed maturation [9]. In *ttg1* mutant Arabidopsis lines complemented with *CsWD40*, the persistent dephosphorylation of CsWD40 may disturb regular plant metabolism, and consequently impact the accumulation process of proanthocyanidins during pod development. The phosphorylation and dephosphorylation of CsWD40 had no significant impact on trichome formation during Arabidopsis plant development. After treatment with MeJA, the main roots of transgenic Arabidopsis with CsWD40^3D^ got longer, indicating that the transgene exhibited insensitivity to MeJA and increased tolerance to stress conditions. Combined with the expression patterns of lignin and flavonoid pathway genes, it is speculated that the phosphorylation of CsWD40 altered carbon allocation in tea plants under drought stress, reducing flavonoid accumulation and enhancing lignin biosynthesis (Fig. S15, see online supplementary material). Our previous research indicated that lignin biosynthesis enhanced resistance to abiotic stress in tea plants [40]. This study provides new insights into how tea plants regulate metabolic fluxes to adapt to or withstand drought stress by modulating the protein phosphorylation signaling pathway. It also further clarifies the regulatory mechanisms of flavonoid synthesis in tea plants under drought stress. The identification of the three phosphorylation sites on CsWD40 provides potential targets for gene editing to develop low astringency cultivars. Cheruiyot *et al.* suggested that polyphenols could serve as potential indicators for assessing the drought tolerance of tea cultivars [41]. Based on the findings of this study, we further propose the potential use of catechin levels to evaluate the degree of drought stress on tea plants in plantations.

## Materials and methods

### Plant materials and growth conditions

The leaves from *C. sinensis ‘Shuchazao*’ were utilized to clone genes, and one-year-old cuttings were subjected to drought treatment. Gene transformation was conducted using Arabidopsis of mutants *ttg1* (CS3099, CsWD40-null mutant) within the Columbia (Col-0) background, and all transgenic plants were produced via agrobacterium-mediated transformation. The plants were grown in a phytotron under cultivation conditions of 16 hours of light, 8 hours of darkness, a temperature of 25 ± 3°C, and a light intensity of 150–200 μmol m^−2^ s^−1^.

### Drought treatment of tea plants

Newly grown shoots were harvested from the tea plants in the plantation. The tea shoots were cultured on ½ MS medium for 24 hours, then they were transferred to ½ MS medium supplemented with 20% PEG for 8, 12, 24, and 48 hours to induce drought stress. In the control group, tea shoots were cultured on ½ MS medium. The two-year-old tea plants were subjected to natural drought conditions. After acclimating in the phytotron for one week, irrigation was suspended, and the plants were maintained without water until leaf wilting occurred.

### Site-directed mutagenesis

Site-directed mutagenesis was performed with an overlap extension PCR [42]. Based on the report, the mutant CsMPK4a undergoes a site modification from -GATTTCATGACTGAG- to -GGTTTCATGACTGCG-, resulting in the formation of a constitutively active form CA-CsMPK4a. Using the same approach, CsWD40 mutant variants *CsWD40^S216A^*, *CsWD40^T221A^*, *CsWD40^S253A^*, *CsWD40^S216A/T221A/S253A^*(*WD40^3D^*), *CsWD40^S216D^*, *CsWD40^T221D^*, *CsWD40^S253D^*, *CsWD40^S216D/T221D/S253D^* (*WD40^3A^*) were constructed. The primer sequences specifically designed for mutagenesis are listed in Table S1 (see online supplementary material).

### Yeast two-hybrid assay

The open reading frames (ORFs) of *CsWD40, CsMPK4a, CA-CsMPK4a, CsMYB5a, CsTT8, CsAN2, CsWD40^3A^*, and *CsWD40^3D^* were amplified and cloned into pGADT7 (Clontech), while the coding sequences of *CsWD40, CsMPK4a, CA-CsMPK4a, CsMYB5a, CsTT8, CsAN_2_, CsWD40^3A^*, and *CsWD40^3D^* were amplified and cloned into pGBKT7 (Clontech).

### Gene cloning and construction of binary vectors for plant transformation

The open reading frames (ORFs) of *CsWD40* and *CsMPK4a* were amplified using gene-specific primers (Table S1, see online supplementary material), and the corresponding mutant cDNAs were generated through site-directed mutagenesis as described above. Subsequently, the amplified ORFs were cloned into pDONR207 vector via BP recombinase (Invitrogen, Life Technologies, Waltham, MA, USA), followed by subcloning into pCB2004 vector using LR recombinase (Invitrogen, Life Technologies) for Arabidopsis transformation. In addition, the ORF of *CsWD40* was amplified and further subcloned into pCambia 1305 vector and pUC-SPYNE vector fused with green fluorescent protein (GFP) and yellow fluorescent protein (YFP), respectively. The constructs of *CsWD40* were genetically transformed by *Agrobacterium tumefaciens* (strain GV3101) to complement the Arabidopsis mutant *ttg1* by flower impregnation.

### Expression and purification of recombinant protein

The ORF of *CsWD40* was cloned into the pMAL-c2X expression vector (Addgene, MA, USA) with a maltose-tag, using Hind III and EcoRI restriction sites. Similarly, the ORF of *CsMPK4a* was cloned into the pRSFDuet™ expression vector (Novagen, Darmstadt, Germany) with a His-tag, using BamHI and PstI restriction sites. The ligation process was carried out using a T4-ligase system and confirmed by sequencing. Subsequently, recombinant plasmids were transformed into *E. coli* Novablue (DE3) competent cells (Novagen, Schwalbach, Germany) via the manufacturer’s protocol. The induction expression and purification of recombinant proteins CsWD40 and CsMPK4a was conducted following the manufacturer’s instructions. Finally, 12% SDS polyacrylamide gel electrophoresis was used to analyse the purified recombinant proteins, which were subsequently detected using commercial antibodies specific to the MBP and His tags.

### Preparation of antibody against phosphorylated CsWD40 peptides

The phosphorylated antibodies specific for phosphorylated CsWD40 were prepared by GL Biochem Ltd (Shanghai, China). The preparation of phosphorylated antibodies against site of WD40^S216^, WD40^T221^, WD40^S253^ were described in the previous work [43]. The peptide sequence used for antibody preparation was listed in Table S2 (see online supplementary material).

### Protein extraction and immunoblot analysis

Bud and first leaf of tea tissues were ground in liquid nitrogen and total protein extracted with protein extraction buffer (50 mM Tris–HCl, pH 7.6, 100 mM NaCl, 2 mM EDTA, 0.5% Triton X-100, 0.5% SDS, 5% glycerol, 2.5 mM DTT, Phenylmethylsulfonyl fluoride (PMSF) and Phosphate Protease inhibitor). Protein concentration was measured with Coomassie Brilliant Blue. Immunoblotting was performed following standard procedures. The antibodies used were as follows: anti-CsWD40 (1:1000 dilution), anti-pSer216 (1:1000 dilution), anti-pThr221 (1:1000 dilution), and anti-pSer253 (1:1000 dilution), all rabbit antibodies.

### Kinase assay *in vitro* and identification of phosphorylation products

For the *in vitro* kinase assay, a mixture containing 6 μg of recombinant CsWD40 protein and 20 μg of recombinant CA-MPK4a protein was prepared in a total volume of 50 μL of kinase reaction buffer. The kinase reaction buffer, with a pH of 7.5, consisted of 50 mM Tris–HCl, 20 mM MgCl_2_, 1 mM EGTA, 1 mM DTT, and 1 mM ATP. The mixture was then incubated at 37°C for 30 minutes. To stop the reaction, 5× SDS loading buffer was added, followed by incubation at 100°C for 5 minutes. The resulting reaction products were separated on a 12% SDS-polyacrylamide gel. To identify the phosphorylation sites on the CsWD40 protein, the reacted solution was also subjected to analysis using the Q Exactive LC–MS/MS System (Thermo Scientific, Waltham, Massachusetts, USA). MS/MS spectra analysis was performed to identify and characterize the phosphorylation products on the CsWD40 protein.

### Proanthocyanidin detection in plant tissue using DMACA staining

The proanthocyanidin in Arabidopsis seed coat was stained by reagent 4-dimethylaminocinnamaldehyde (DMACA) according to reference [39]. Approximately 100 dry Arabidopsis seeds were gently washed with water in a 1.5 mL centrifuge tube. The seeds were then incubated with 1 mL of the DMACA reagent, which contained 2% (w/v) DMACA in 3 M HCl and 50% (w/v) methanol, at room temperature in the dark for 12 hours. After the incubation, the seeds were washed three times with 75% (v/v) ethanol to remove any excess DMACA reagent. The treated seeds were immediately photographed.

### Extraction and detection of proanthocyanidins and anthocyanins

To extract and detect proanthocyanidins and anthocyanins from Arabidopsis, phenol extraction was performed as follows. Approximately 0.02 g of Arabidopsis green pod or 0.2 g of Arabidopsis seedling was weighed accurately and placed into a 2.0 mL centrifuge tube with two small steel balls. The tubes were frozen in liquid nitrogen, and the pods were ground into a fine powder using a pre-cooled ball mill. Then, 500 μL of the extraction solvent (consisting of 80% methanol, 0.2% HCl, and 19.8% H_2_O) was added to the tube. The mixture was subjected to ultrasonication in ice water at 70 Hz for 10 minutes and was mixed upside down every 4 minutes. The supernatant was then collected in a new 2.0 mL centrifuge tube by centrifugation at 10000 r/min for 10 minutes at 4°C. The residue was extracted repeatedly 4–5 times, and the resulting supernatants were combined and the total volume was adjusted to 2 mL.

The levels of proanthocyanidins in Arabidopsis seeds were quantified using the DMACA reagent as a reporter [44]. To determine the relative content of proanthocyanidins, a sample extract containing proanthocyanidins (150 μL) was mixed with 500 μL of DMACA reagent (0.2% w/v DMACA in methanol-3 M HCl) in a colorimetric dish. For blank samples, an additional 150 μL of methanol-3 M HCl was used instead of the sample extract. The mixture was allowed to react at room temperature for 15 minutes, and the optical density (OD) value was immediately measured at 640 nm using a spectrophotometer. The resulting OD value represents the relative content of proanthocyanidins present in the sample.

To determine the levels of anthocyanin, a seedling extract (500 μL) was combined with water (500 μL) and chloroform (300 μL). The resulting mixture was thoroughly shaken to ensure proper mixing. Subsequently, centrifugation was performed at 6000 rpm/min and 4°C for 10–15 minutes using a rotor with a diameter of 10 cm. After centrifugation, the extract supernatant was collected, and its OD value was measured at a wavelength of 530 nm. The resulting OD value represents the relative content of anthocyanin present in the sample.

### Protein subcellular localization and interaction

Protoplast isolation and transfection assays were performed for Arabidopsis and tea leaf using established protocols. PEG-mediated protoplast transformation of Arabidopsis was performed according to the reference [45].

The first leaves (7–9 days after germination) were used for tea protoplast transformation. The extraction and transformation of tea protoplasts can be conducted using a method similar to that of Arabidopsis [45]. Both sets of protoplasts were transfected with DNA constructs of interest using PEG-mediated transfection and analysed using appropriate techniques. The subcellular localization of CsWD40, CsWD40^S216A^, CsWD40^T221A^, CsWD40^S253A^ was analysed using the pUC19 vector. The pUC19 vector carries a GFP tag and is driven by the CaMV 35S promoter. Primers used in constructing the above vector were shown in Table S1 (see online supplementary material).

### BiFC assay in tea and Arabidosis protoplasts

The bimolecular fluorescence complementation (BiFC) assay can be used to detect protein–protein interactions in Arabidopsis protoplasts. This method utilizes the pUC-SPYNE and pUC-SPYCE vectors, which contain yellow fluorescent protein (YFP) and are fused at the nitrogen and carbon termini, respectively. For detailed information on these vectors and their usage, please refer to the literature [46]. The open reading frame of the gene was transferred from the entry vector into the multiple cloning sites of pUC-SPYNE/pUC-SPYCE vector using XbaI and BamHI restriction enzyme cleavage sites. The primers used for constructing the aforementioned vectors are listed in Table S1 (see online supplementary material).

Protein–protein interactions were detected through fluorescence using confocal microscopy, with the presence of fluorescence indicating interaction, while its absence suggests no interaction.

### Gene transient expression in tea leaves

Two-year-old cutting seedlings of tea plants were utilized for transient gene expression of *CsMPK4a* (*Ca-CsMPK4a*), *CsWD40^3D^*, *CsWD40^3A^*. The *A. tumefaciens* suspension was prepared using the conventional method for infecting plants. Wounds were inflicted on both sides of the tea leaf veins, and the suspension was injected into the leaf tissue using a 5 mL syringe. The experimental group suspension and control group suspension were separately injected into the two sides of the leaf veins. The plants were then incubated in a greenhouse for 12 hours, and *Colletotrichum* culture plates were applied to the injection sites to inoculate plants. The entire tea plant was wrapped with transparent film and sprayed with water daily to maintain high humidity. After the additional 48–36 hours, the pink ring phenotype was observed, and samples were collected for analysis. The symptoms of the leaf lesions were recorded using a stereo microscope (ZEISS, Germany). Gene expression levels were detected using quantitative real-time PCR analysis. The real-time PCR reaction was performed according to the manufacturer’s instructions. The data was normalized based on the expression levels of the reference gene GAPDH. The gene expression level was calculated by the equation Y = 10^ΔCT/3^ × 100%. △C_T_ = the mean Ct value of the reference gene − the mean Ct value of target gene. The area of pink ring around the lesion was determined using ImageJ software. The relative area of the pink ring is defined as the area of the pink ring minus the area of necrotic lesions. More than four biological replicates were used in this experiment.

### Statistical analysis

Statistical analysis was conducted using SPSS software (Version 16.0, SPSS Inc., Chicago, IL, USA). To assess trends, Tukey’s test was used for multiple comparative analyses, and significant differences were indicated by distinct lowercase letters. A *P* value of less than 0.05 was considered statistically significant. Two-group comparisons were performed using Student’s *t*-test, and significant differences were denoted by one asterisk (*P* < 0.05) or two asterisks (*P* < 0.01). Data were obtained from at least three independent biological replicates.

### Accession numbers

Sequence data from this article can be found at Tea Genome Platform: http://139.196.163.62/. Accession numbers of genes in the Tea Genome Platform are listed in Table S3 (see online supplementary material).

## Supplementary Material

Web_Material_uhae136
