# Supplementary material for: The phosphorylation of a WD40-repeat protein negatively regulates flavonoid biosynthesis in Camellia sinensis under drought stress
Source: Hortic Res. 2024 May 5;11(7):uhae136. doi: 10.1093/hr/uhae136 (PMC11237189; doi:10.1093/hr/uhae136)
Supplement: Web_Material_uhae136 [file web_material_uhae136.zip › 4.25 Supplementary information.docx]

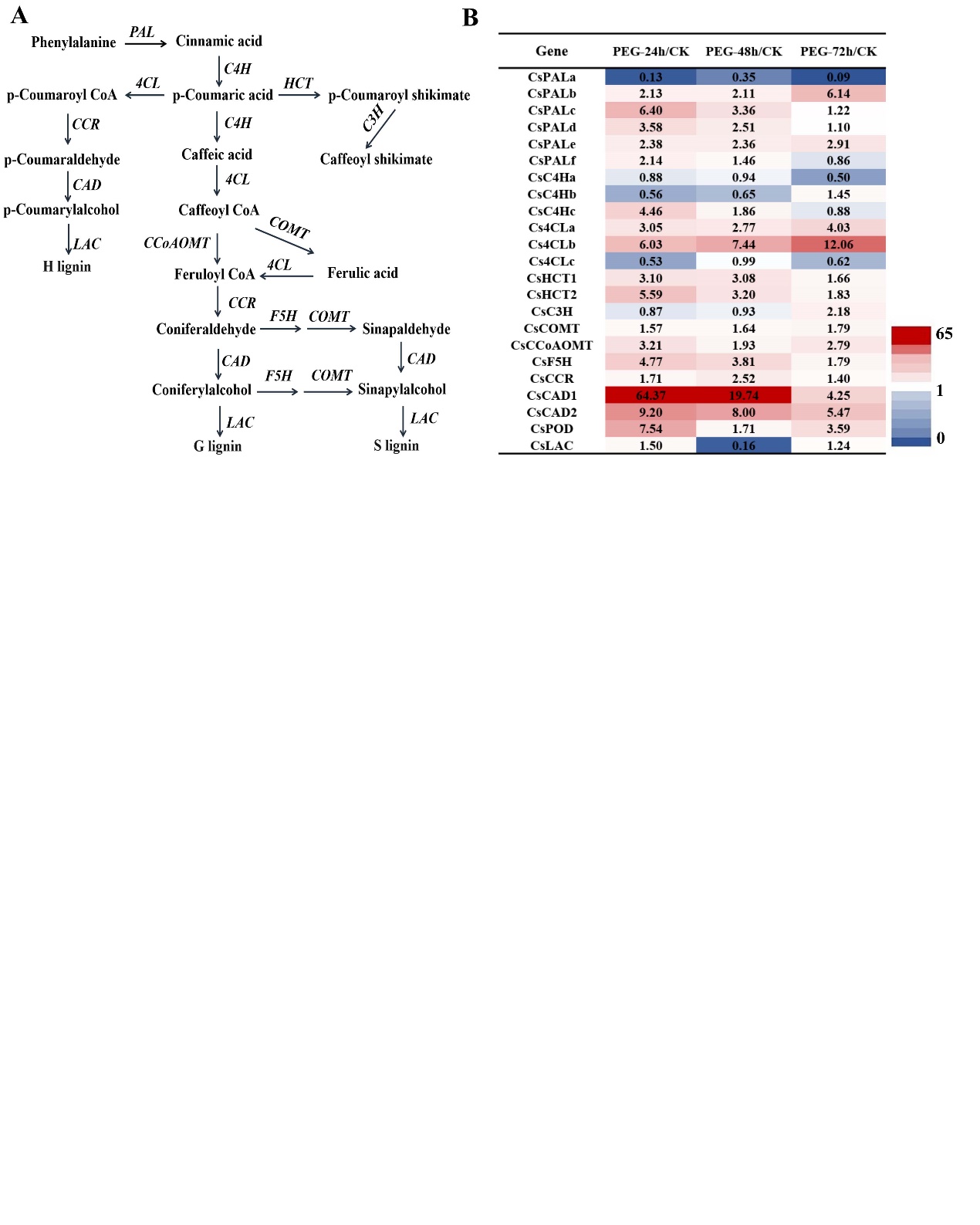


**Figure S1. Gene expression profiles of lignin biosynthesis in the tea plant under drought stress simulated by polyethylene glycol 4000.**

A. Schematic diagram of biosynthesis pathways of lignin in the plant. B. The relative expression level of genes in lignin biosynthesis pathway of tea plant under drought stress by simulation of 20%PEG4000 for 24 h, 48 h, 72 h. The FPKM value of gene expression at different time was list in Supplemental data 5.


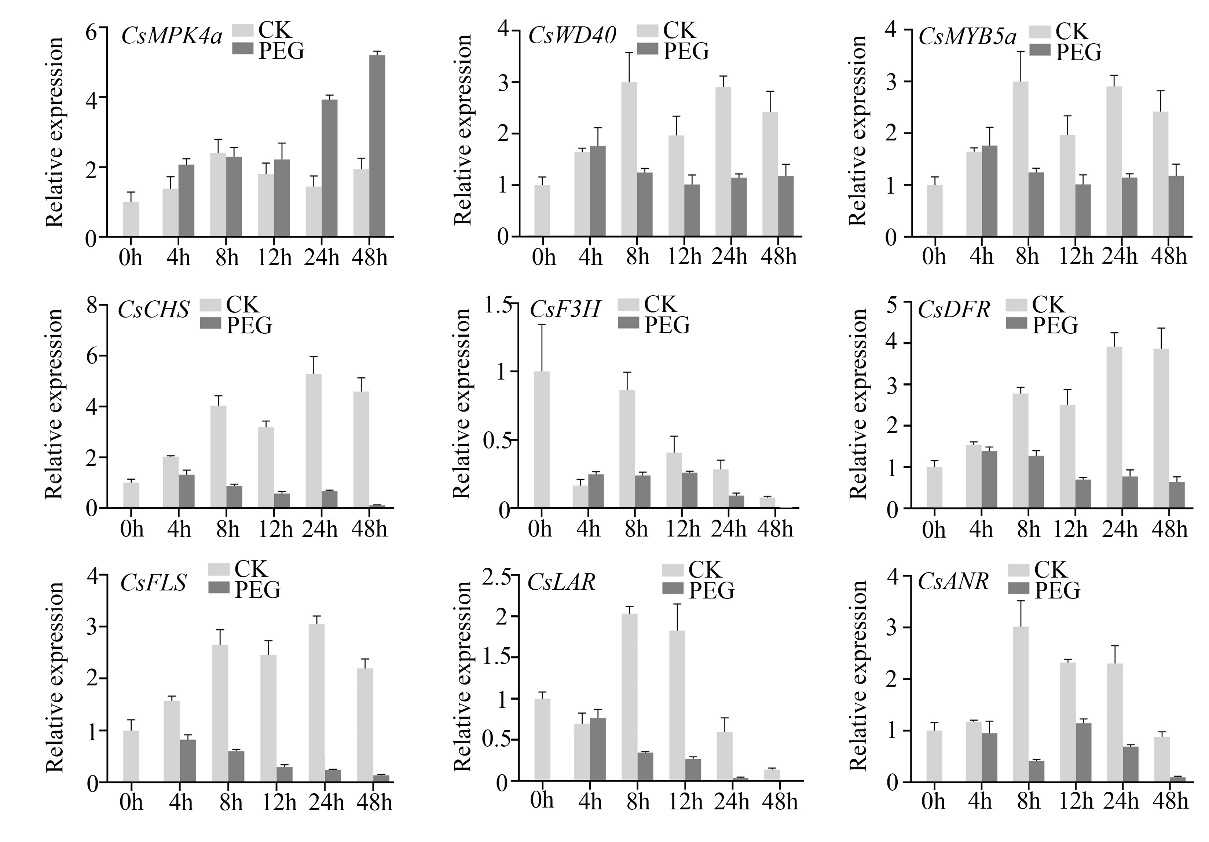


**Fig.S2 The key gene expression profiles involved in flavonoid biosynthesis pathway of tea plant under drought stress by simulation of 20%PEG4000 for 0-48 h.**

Note: The gene expression was detected by qRT-PCR.


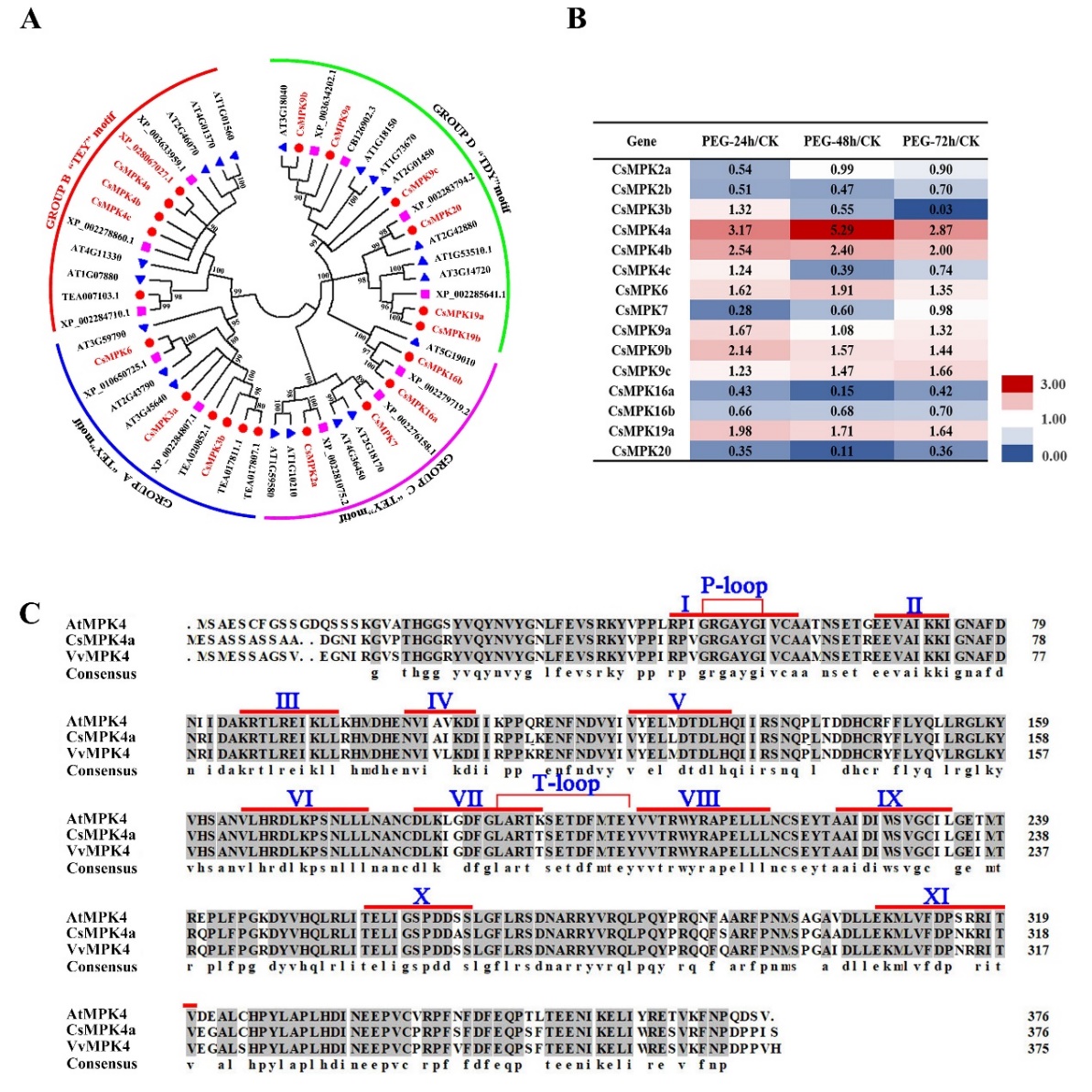


**Figure S3.** **Effects of drought stress on the expression of genes in MPK family in *C.sinensis* leaves.**

A. Phylogenetic tree analysis of CsMPK and other plant MPK families. B. Gene expression profiles of MAPK family in the tea plant under drought condition simulated by polyethylene glycol. C. The alignment of CsMPK4a protein sequence with the orthologous protein from *A. thaliana* and *Vitis vinifera*.


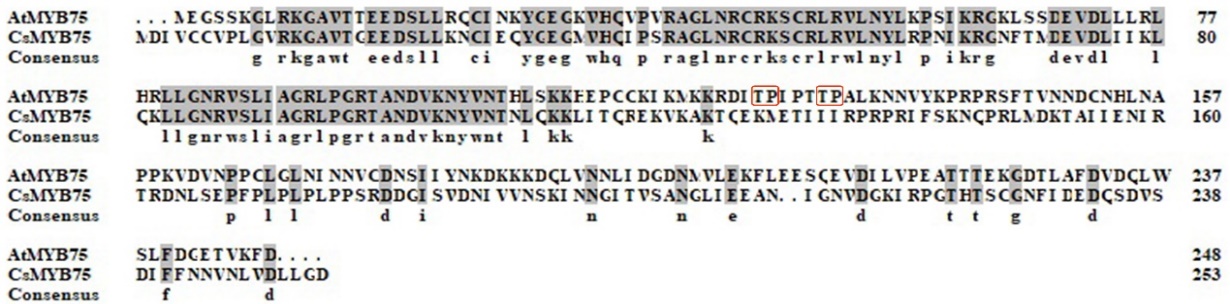
**Figure S4. Protein sequence alignment between CsMYB75 from *C.sinensis* and AtMYB75 from *A. thaliana.***
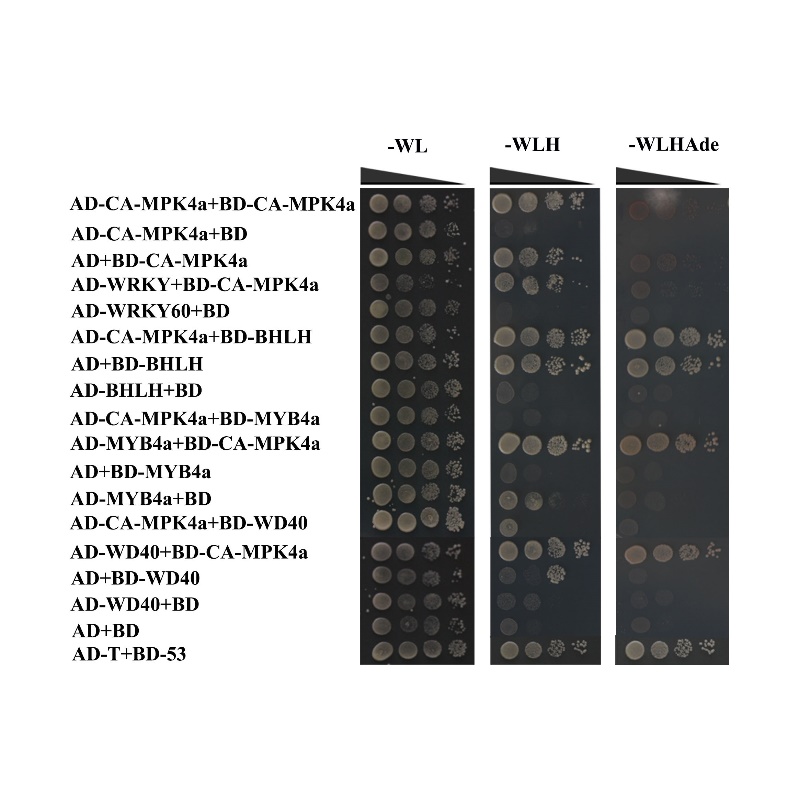


**Figure S5. Interaction of CsMPK4a with proteins regulating the flavonoid biosynthesis by Yeast two-hybrid and dual luciferase assay**

CA-MPK4a：The formation of a constitutively active form CsMPK4a. Refer to the Methods section for details on the gene mutation method.


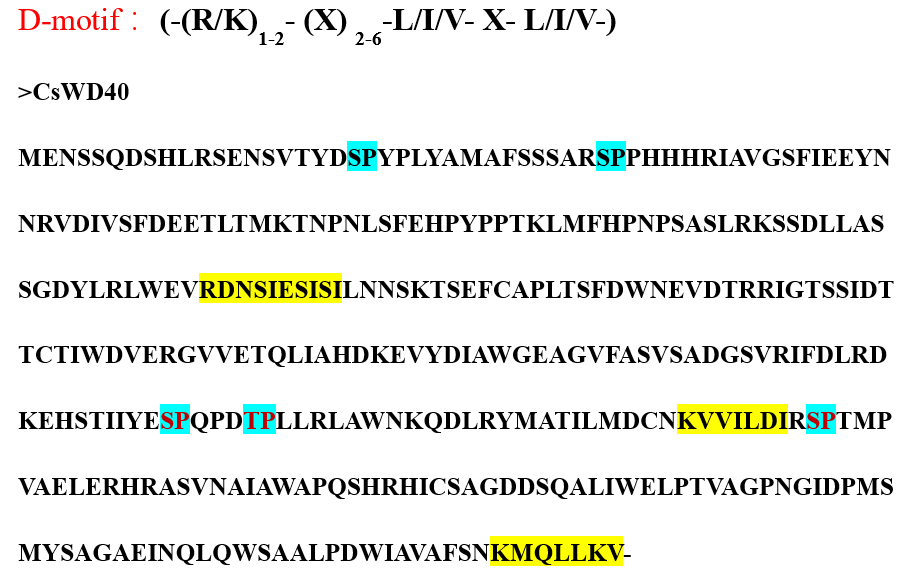


**Figure S6. Prediction of CsMPK4a d-motif domain and phosphorylation sites on CsWD40.**

The yellow box marks the d-motif domain of CsWD40 protein; Blue marks are the predicted potential phosphorylation sites of CsMPK4a


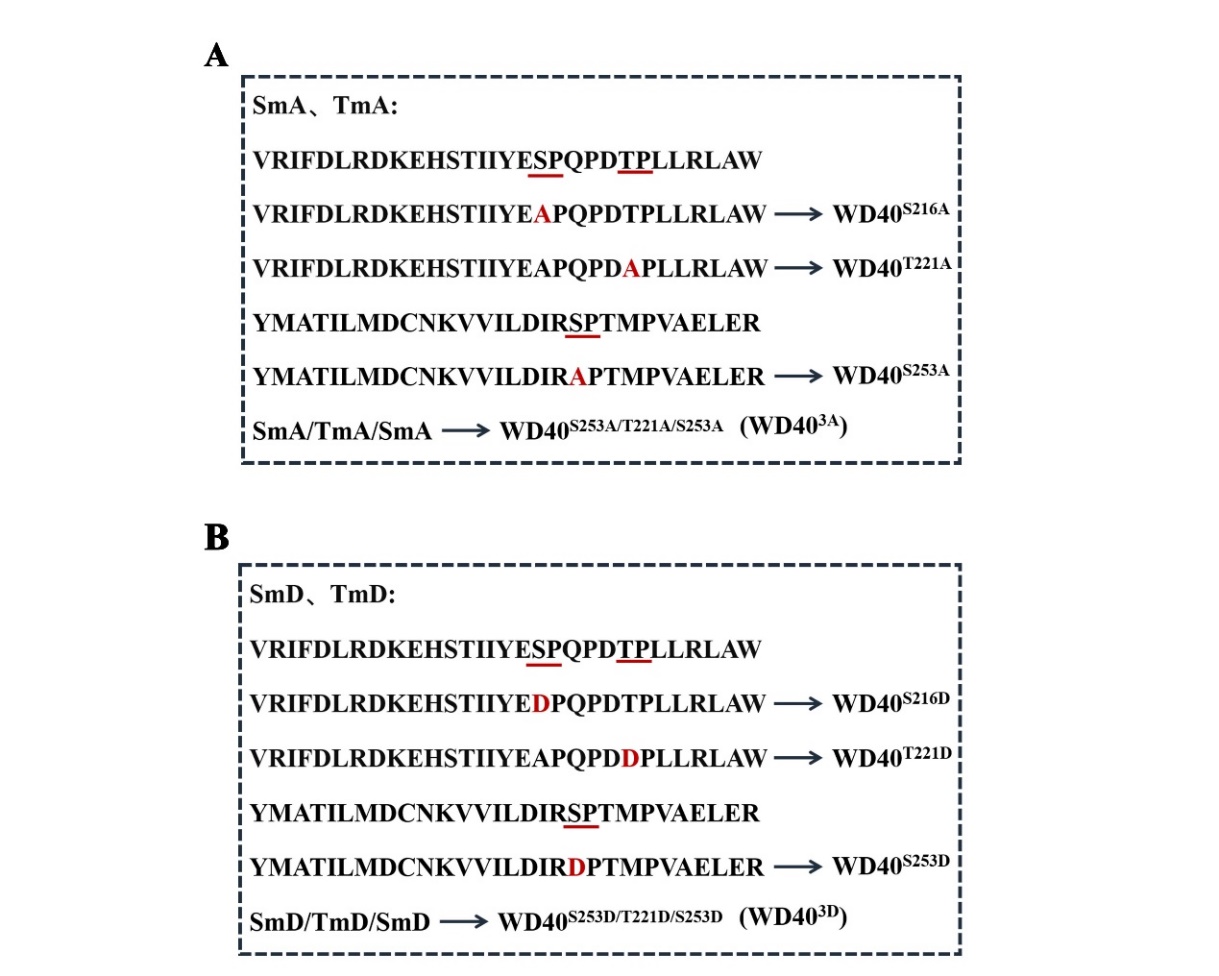


**Figure S7. Diagram of CsWD40 dephosphorylation (A) and sustained phosphorylation site mutation (B).**


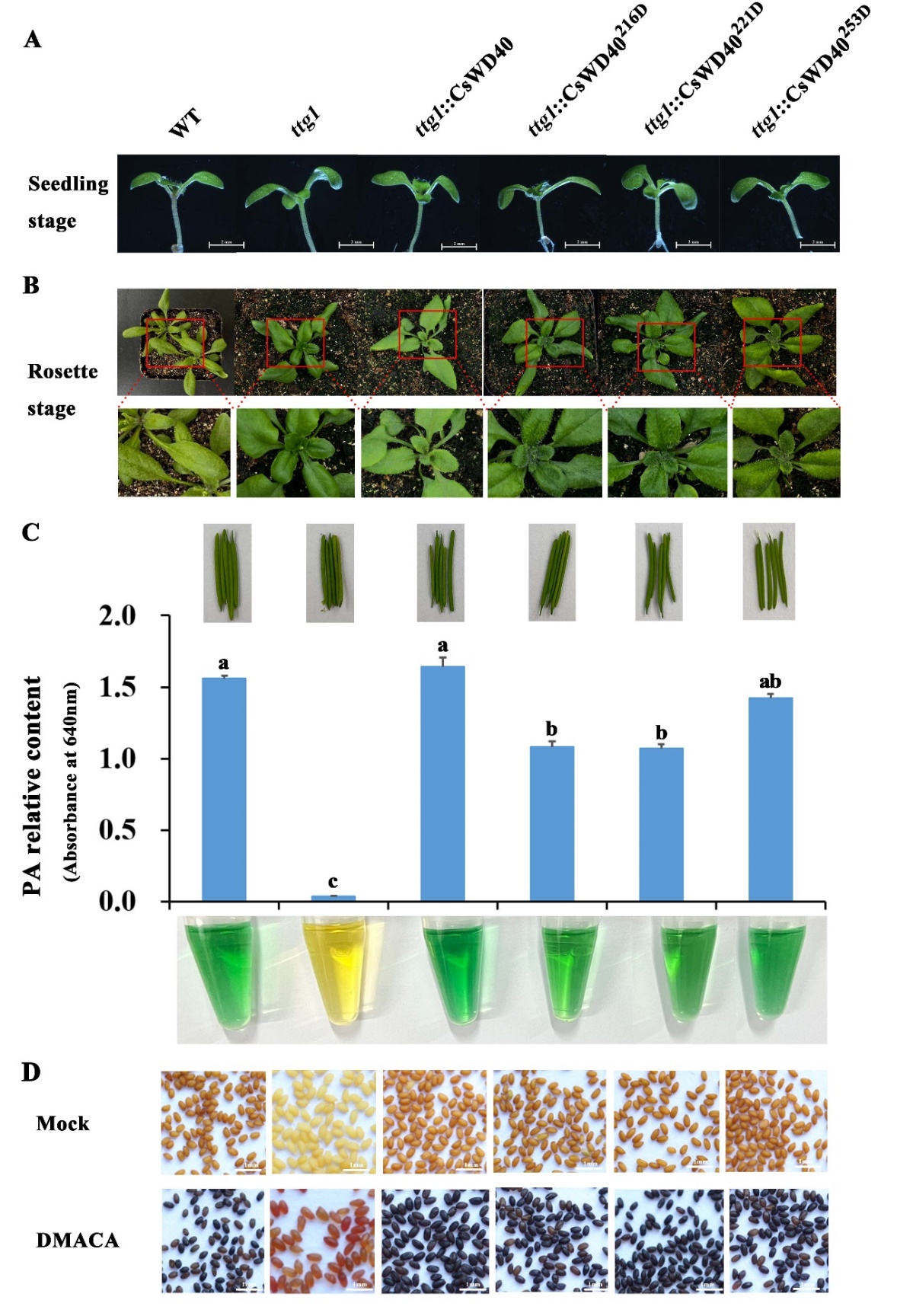


**Figure S8. Trichome and proanthocyanidin accumulation phenotype of CsWD40 single-site phosphorylated transgenic Arabidopsis.**

A. Growth phenotype of seedlings during cotyledon expansion stage. B. Trichome phenotype of transgenic *A. thaliana* with 2-4 true leaves at the rosette stage. C. Content analysis of proanthocyanidin in green pod of transgenic *A. thaliana*. D. Seed coat phenotype and DMACA staining phenotype of mature Arabidopsis seeds.


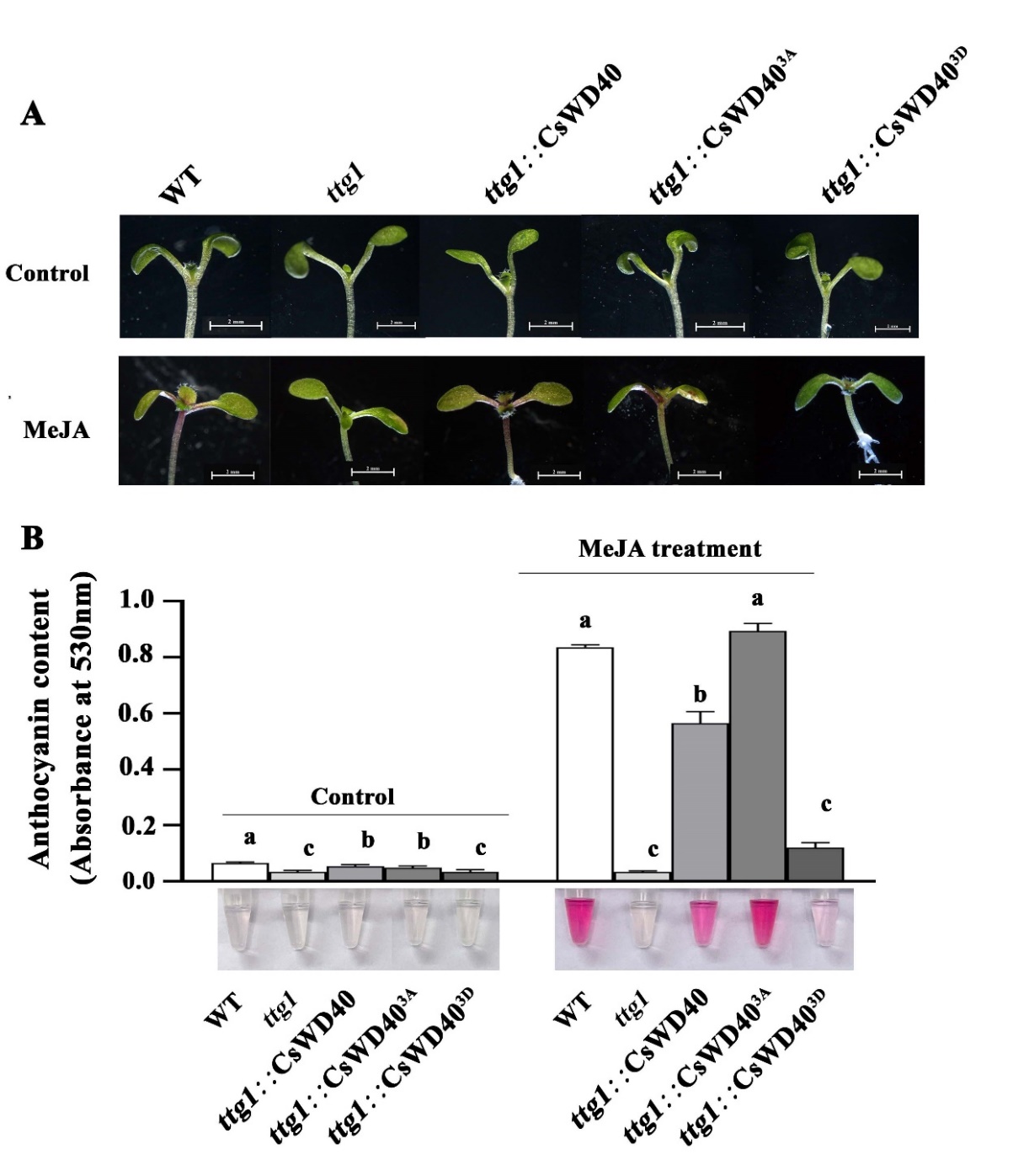


**Figure S9. Anthocyanin content of tri-site** **phosphorylated and dephosphorylated CsWD40 transgenic *A. Thaliana.***

A. Growth and development phenotypes of *ttg1*::*CsWD40*^3A^ and *ttg1*::*CsWD40*^3D^ transgenic Arabidopsis on medium with and without methyl jasmonate addition at seedling stage. B. Anthocyanin content of *ttg1*::*CsWD40*^3A^ and *ttg1*::*CsWD40*^3D^ transgenic Arabidopsis on medium with and without methyl jasmonate addition.


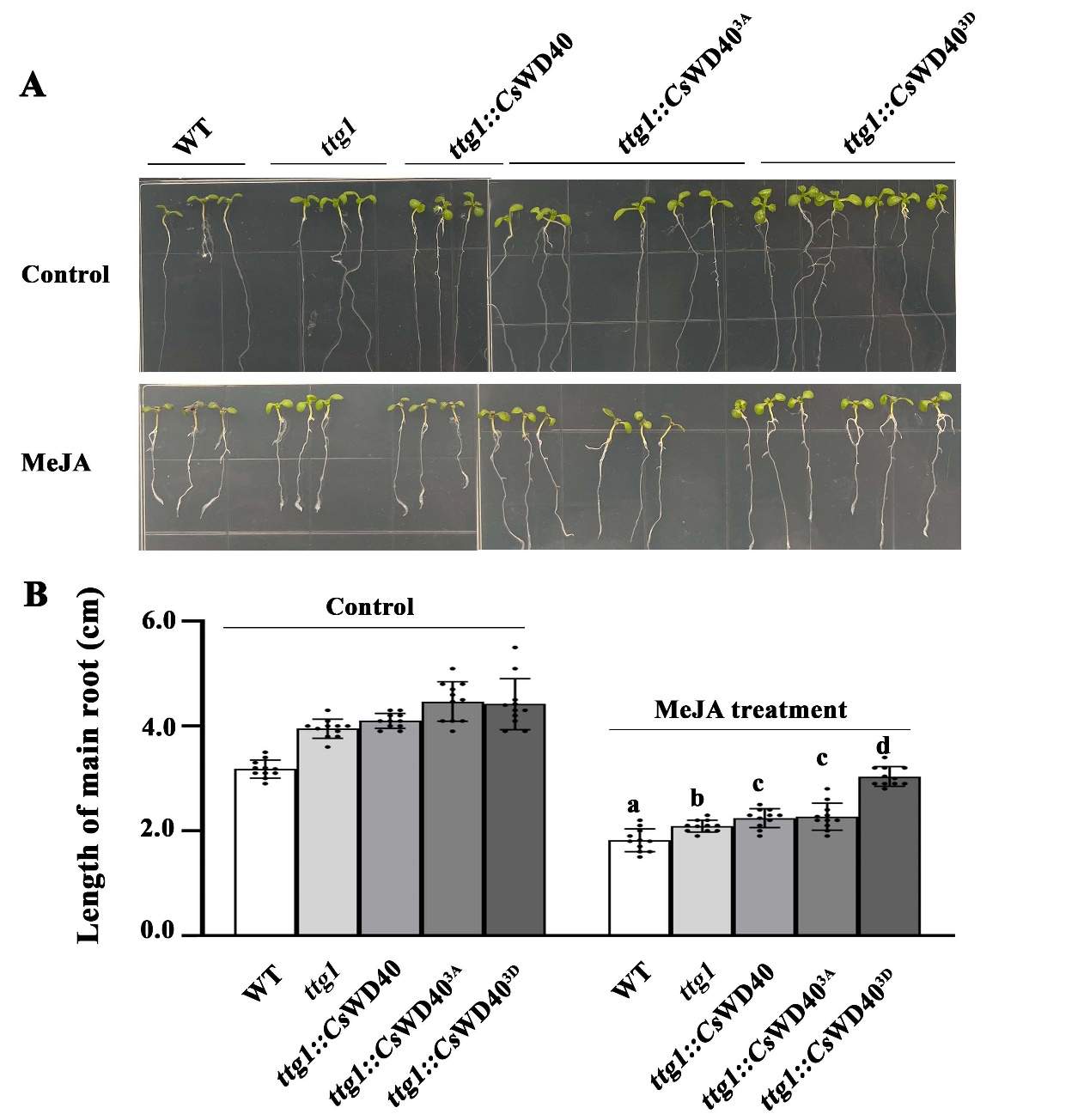


**Figure S10. The root development phenotype of tri-site phosphorylated and dephosphorylated CsWD40 transgenic *A. Thaliana.***

A and B. The main root development phenotype and length of dephosphorylated and phosphorylated CsWD40 complementary mutants Arabidopsis *ttg1*::*CsWD40*^3A^ and *ttg1*::*CsWD40*^3D^ on medium containing methyl jasmonate.


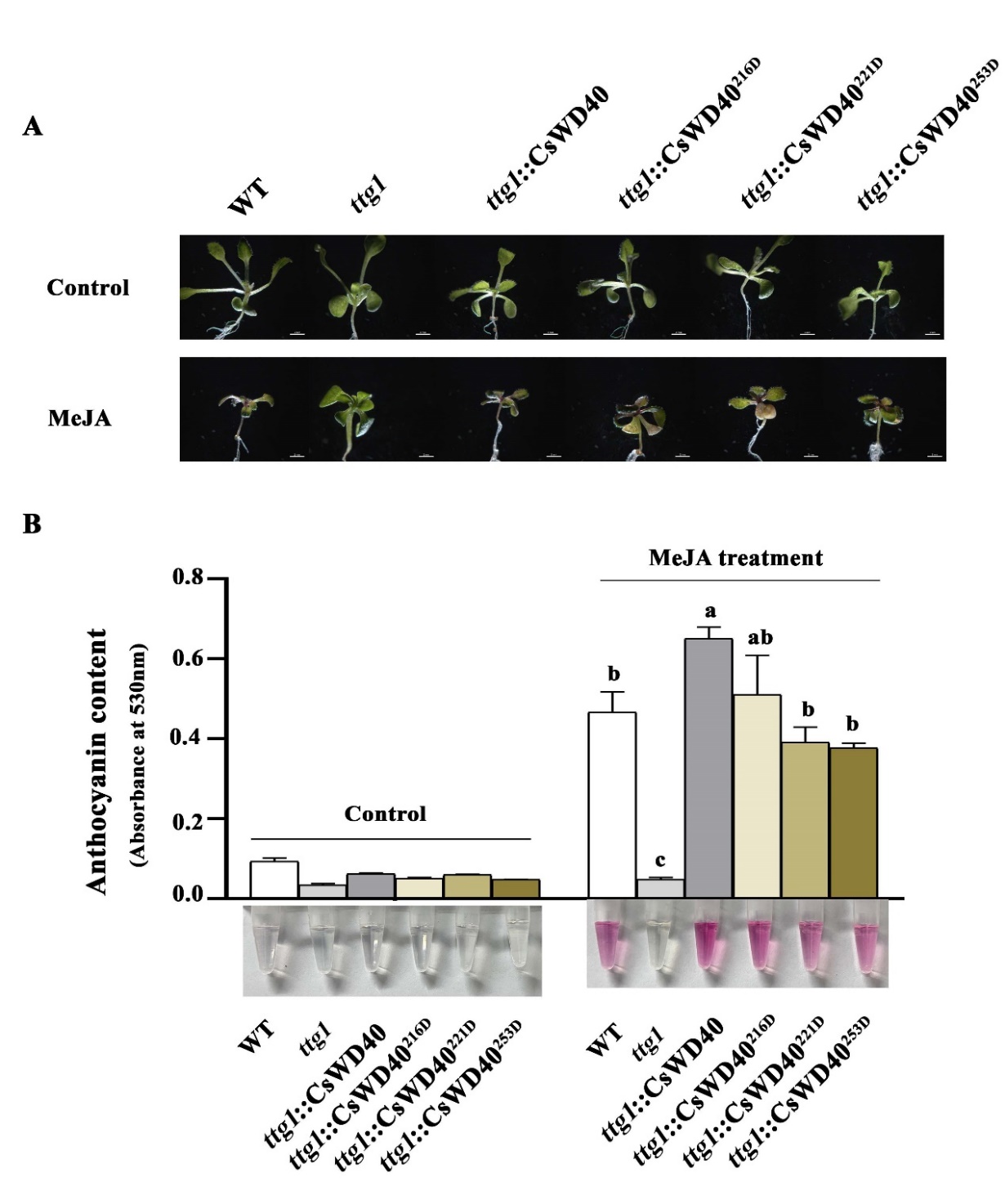
**Figure S11. Phenotype and anthocyanin analysis of CsWD40 transgenic *A. Thaliana* with a single site phosphorylation under MeJA stress.**

A. Growth and development phenotypes of *ttg1*::*CsWD40*^216D^, *ttg1*::*CsWD40*^221D^ and *ttg1*::*CsWD40*^253D^ transgenic Arabidopsis on medium with and without methyl jasmonate addition at seedling stage. B. Anthocyanin content of *ttg1*::*CsWD40*^216D^, *ttg1*::*CsWD40*^221D^ and *ttg1*::*CsWD40*^253D^ transgenic Arabidopsis on medium with and without methyl jasmonate addition.


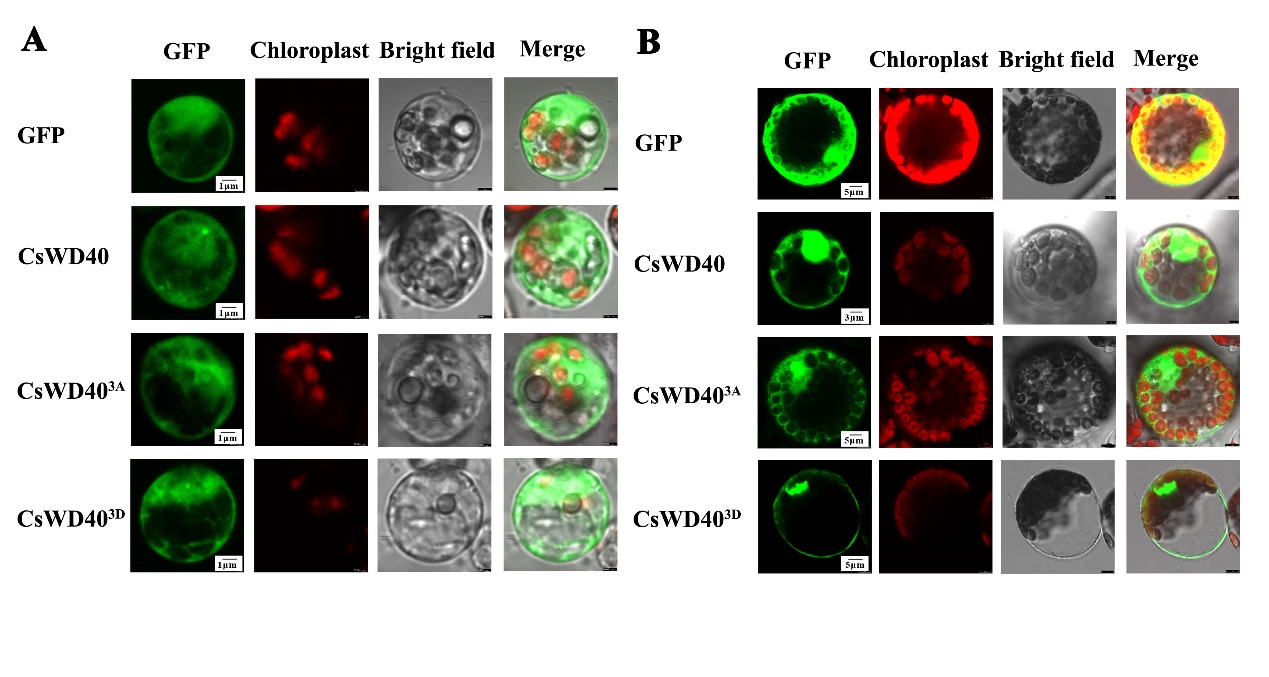


**Figure S12. Subcellular localization of CsWD40, CsWD40^3A^ and CsWD40^3D^ .**

A and B. Localization of CsWD40, CsWD40^3A^ and CsWD40^3D^ in tea (A) and Arabidopsis protoplasts (B).


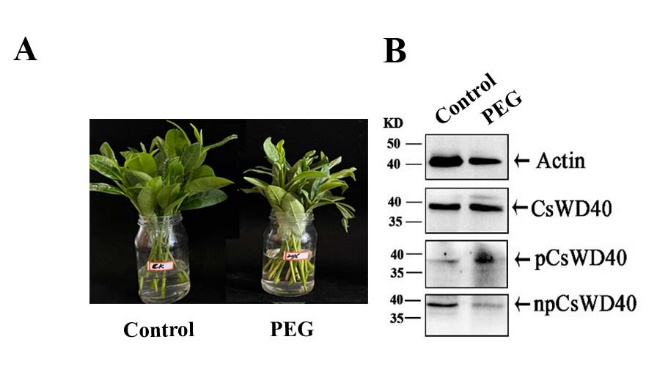


**Figure S13. Phosphorylation levels of CsWD40 in leaves treated by 20% PEG for 48 hours.**

A. The phenotype of tea shoots under drought treatment simulated by 20% PEG for 48 h. B. Analysis of the abundance of CsWD40 protein, phosphorylated CsWD40 protein, and dephosphorylated CsWD40 protein between the drought-treated group and the control group. A specific mixture of three site-specific antibodies was utilized for the immunological analysis of phosphorylated pCsWD40, whereas a distinct mixture of three site-specific antibodies was applied for the analysis of non-phosphorylated npCsWD40.


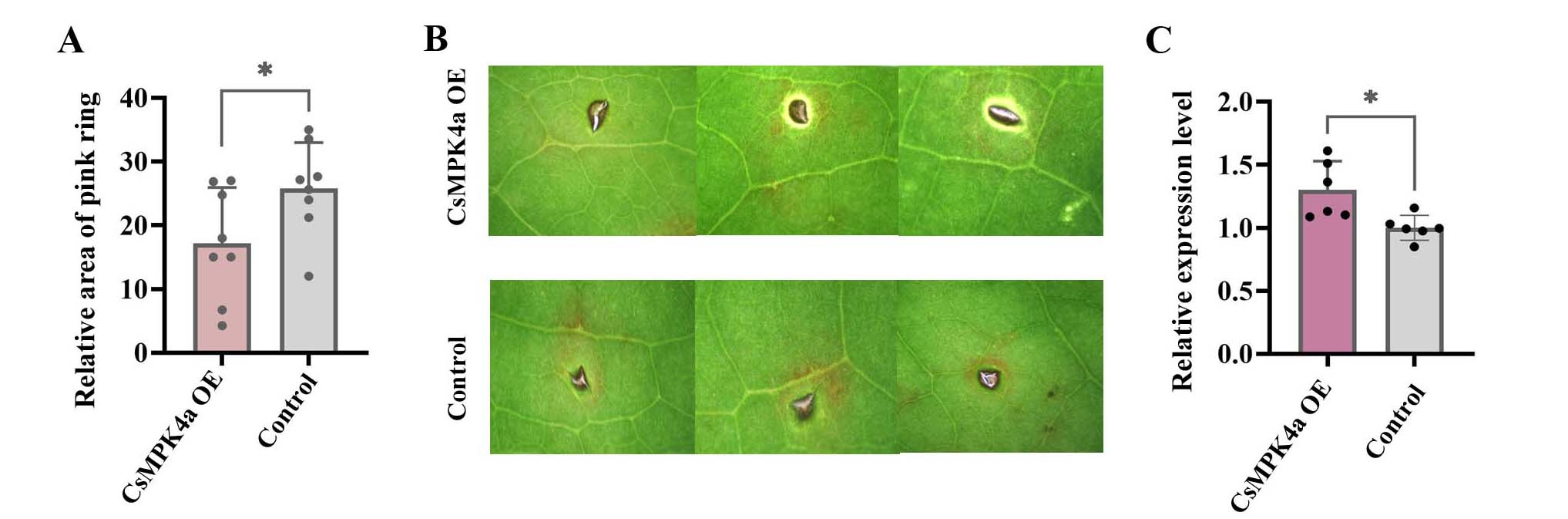


**Figure S14. Effect of transient overexpression of *CsMPK4a* on anthocyanin biosynthesis in tea leaves under stress.**

A. Transgene verification through *CsMPK4a* gene expression level detection by qRT-PCR. B. The effect of overexpressing the *CsMPK4a* gene on the Colletotrichum-induction of anthocyanin accumulation in plants. The *CA-CsMPK4* mutant gene was utilized to infect tea leaves, enabling expression of the phosphorylation-active CsMPK4a protein. C. The relative area of pink ring in control group and treatment group of transient overexpression of *CsMPK4a*.


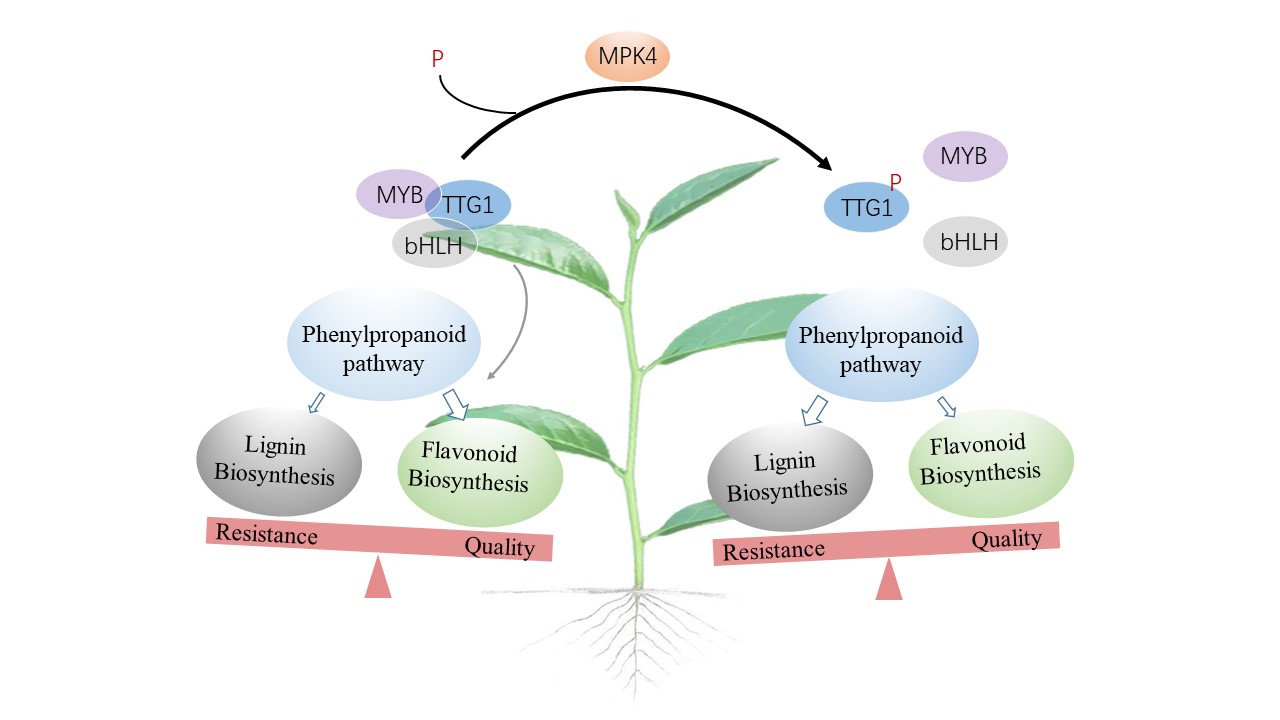


**Figure S15. Speculative mechanisms of CsWD40 phosphorylation altering carbon allocation in tea plants under drought stress, reducing flavonoid accumulation and enhancing lignin biosynthesis.**

| **Supplementary Table S1. primers used in this study** | | |
| --- | --- | --- |
|  | primer name | primer sequence(5’-3’) |
| Site-directed mutagenesis | CA-CsMPK4a-F | CATCTGAAACAGGTTTCATGACTGCGTATGTTGTTAC |
|  | CA-CsMPK4a-R | GTAACAACATACGCAGTCATGAAACCTGTTTCAGATG |
|  | CsWD40-ORF-F | ATGGAGAATTCGAGCCAAG |
|  | CsWD40-ORF-R | TCAAACTTTCAGAAGCTGCA |
|  | CsWD40-216D-F | CTCTACCATTATATACGAGGATCCTCAACCGGACACTCC |
|  | CsWD40-216D-R | GGAGTGTCCGGTTGAGGATCCTCGTATATAATGGTAGAG |
|  | CsWD40-221D-F | GAGTCCTCAACCGGACGATCCTTTGCTTAGATTGGCTTGG |
|  | CsWD40-221D-R | CCAAGCCAATCTAAGCAAAGGATCGTCCGGTTGAGGACTC |
|  | CsWD40-253D-F | GTGATCTTGGATATTCGAGATCCAACAATGCCGGTTGCAG |
|  | CsWD40-253D-R | CTGCAACCGGCATTGTTGGATCTCGAATATCCAAGATCAC |
| Plant transgenic assay | CsWD40-BP-F | GGGGACAAGTTTGTACAAAAAAGCAGGCTATGGAGAATTCG |
|  | CsWD40-BP-R | GGGGACCACTTTGTACAAGAAAGCTGGGTTCAAACTTTCAG |
| Yeast two-hybrid assay | CsWD40-AD-F | ATGGAGGCCAGTGAATTCATGGAGAATTCGAGCCAAG |
|  | CsWD40-AD-R | AGCTCGAGCTCGATGGATCCCTCAAACTTTCAGAAGCTGCA |
|  | CsWD40-BD-F | ATGGCCATGGAGGCCGAATTCATGGAGAATTCGAGCCAAG |
|  | CsWD40-BD-R | CGCTGCAGGTCGACGGATCCCTCAAACTTTCAGAAGCTGCA |
| Protein subcellular localization | CsWD40-pUC19-F | GAGAACACGGGGGACTCTAGA ATGGAGAATTCGAGCCAAG |
|  | CsWD40- pUC19-R | CATGTCGACCTCGAGGGATCCAACTTTCAGAAGCTGCA |
|  | CsWD40-1305-F | CCGGAGCTAGCTCTAGAATGGAGAATTCG |
|  | CsWD40-1305-R | CTTGCTCACCATGGATCCAACTTTCAGAAG |
| BiFC Assay | CsWD40-cLUC-F: | TACGCGTCCCGGGGCGGTACC ATGGAGAATTCGAGCCAAG |
|  | CsWD40-cLUC-R: | ACGAAAGCTCTGCAGGTCGAC TCAAACTTTCAGAAGCTGCA |
|  | CsWD40-nLUC-F: | ACGGGGGACGAGCTCGGTACC ATGGAGAATTCGAGCCAAG |
|  | CsWD40-nLUC-R: | CGCGTACGAGATCTGGTCGAC AACTTTCAGAAGCTGCA |
|  | CsMYB5a-cLUC-F | TACGCGTCCCGGGGCGGTACC ATGGGGAGGAGTCCATGCTGC |
|  | CsMYB5a-cLUC-R | ACGAAAGCTCTGCAGGTCGAC TCATGGCCAGTCCTCAGAATCAAG |
|  | CsMYB5a-nLUC-F | ACGGGGGACGAGCTCGGTACC ATGGGGAGGAGTCCATGCTGC |
|  | CsMYB5a-nLUC-R | CGCGTACGAGATCTGGTCGAC TGGCCAGTCCTCAGAATCAAG |
|  | CsAN2- cLUC-F | TACGCGTCCCGGGGCGGTACC ATGGACATTGTTTGTTGTG |
|  | CsAN2- cLUC-R | ACGAAAGCTCTGCAGGTCGAC TTCATCACCTAACAG |
|  | CsAN2- nLUC-F | ACGGGGGACGAGCTCGGTACC ATGGACATTGTTTGTTGTG |
|  | CsAN2- nLUC-R | CGCGTACGAGATCTGGTCGAC TCATTCATCACCTAACAG |

| **Supplementary Table S2. The peptide used for antibody preparation** | | |
| --- | --- | --- |
| Site Number | Antibody Name | AntigenSequence: |
| S216 | Anti-pS216 | Cys-RDKEHSTIIYE(pS)PQPD |
|  | Anti-S216(NP control) | Cys-RDKEHSTIIYESPQPD |
| T221 | Anti-pT221 | Cys-QPD(pT)PLLRLAWNKQD |
|  | Anti-T221(NP control) | Cys-QPDTPLLRLAWNKQD |
| S253 | Anti-pS253 | DIR(pS)PTMPVAELERH-Cys |
|  | Anti-S253(NP control) | DIRSPTMPVAELERH-Cys |

| **Supplementary Table S3. Accession number of genes in Tea Genome Platform** | |
| --- | --- |
| Gene Number | Accession number in TPIA |
| PALa | TEA023243.1 |
| PALb | TEA003137.1 |
| PALc | TEA024587.1 |
| PALd | TEA034008.1 |
| PALe | TEA014056.1 |
| PALf | TEA003374.1 |
| C4Ha | TEA014864.1 |
| C4Hb | TEA034001.1 |
| C4Hc | TEA016772.1 |
| 4CLa | TEA025906.1 |
| 4CLb | TEA027829.1 |
| 4CLc | TEA034012.1 |
| CHSa | TEA023340.1 |
| CHSb | TEA023333.1 |
| CHSc | TEA034042.1 |
| CHIa | TEA013101.1 |
| CHIb | TEA034003.1 |
| CHIc | TEA033031.1 |
| CHId | TEA018689.1 |
| F3Ha | TEA023790.1 |
| F3Hb | TEA034016.1 |
| DFRa | TEA032730.1 |
| DFRb | TEA023829.1 |
| DFRc | TEA010588.1 |
| F3'H | TEA006847.1 |
| F3'5'Ha | TEA013315.1 |
| F3'5'Hb | TEA026296.1 |
| LARa | TEA027582.1 |
| LARb | TEA026458.1 |
| LARc | TEA021535.1 |
| ANRa | TEA022960.1 |
| ANRb | TEA009266.1 |
| ANSa | TEA010322.1 |
| ANSb | TEA015769.1 |
| FLSa | TEA006643.1 |
| FLSb | TEA010328.1 |
| FLSc | TEA016601.1 |
| MYB5a | TEA004608.1 |
| MYB5b | TEA031375.1 |
| MYB5c | TEA027333.1 |
| MYB5d | TEA014311.1 |
| MYB5e | TEA002308.1 |
| MYB6a | TEA018834.1 |
| MYB6b | TEA011004.1 |
| MYB7a | TEA009412.1 |
| HCT1 | TEA011691.1 |
| HCT2 | TEA032135.1 |
| WD40 | TEA000080.1 |
| GL3 | TEA033721.1 |
| BHLH-TT8 | TEA010741.1 |
| HCT1 | TEA011691.1 |
| HCT2 | TEA032135.1 |
| C3H | TEA016161.1 |
| COMT | TEA011119.1 |
| CCoAOMT | TEA012735.1 |
| F5H | TEA032005.1 |
| CCR | TEA032890.1 |
| CAD1 | TEA001438.1 |
| CAD2 | TEA000393.1 |
| POD | TEA001789.1 |
| LAC | TEA021330.1 |
| MPK2a | TEA031435.1 |
| MPK2b | TEA016315.1 |
| MPK3a | TEA026040.1 |
| MPK3b | TEA020851.1 |
| MPK4a | TEA006436.1 |
| MPK4b | TEA006273.1 |
| MPK4c | TEA021759.1 |
| MPK6 | TEA024415.1 |
| MPK7 | TEA032724.1 |
| MPK9a | TEA012905.1 |
| MPK9b | TEA015676.1 |
| MPK9c | TEA022268.1 |
| MPK16a | TEA026883.1 |
| MPK16b | TEA031053.1 |
| MPK19a | TEA018880.1 |
| MPK20 | TEA004137.1 |
| Note:　Tea Genome Platform : http://139.196.163.62/. ‘TEA’ is the number of SHUCHAZAO1 genome. | |
